# Complete Structural Model of *Escherichia coli* RNA Polymerase from a Hybrid Approach

**DOI:** 10.1371/journal.pbio.1000483

**Published:** 2010-09-14

**Authors:** Natacha Opalka, Jesse Brown, William J. Lane, Kelly-Anne F. Twist, Robert Landick, Francisco J. Asturias, Seth A. Darst

**Affiliations:** 1 The Rockefeller University, New York, New York, United States of America; 2 Department of Cell Biology, The Scripps Research Institute, La Jolla, California, United States of America; 3 Department of Pathology, Brigham & Women's Hospital, Boston, Massachusetts, United States of America; 4 Departments of Biochemistry and Bacteriology, University of Wisconsin, Madison, Wisconsin, United States of America; Cold Spring Harbor Laboratory, United States of America

## Abstract

The *Escherichia coli* transcription system is the best characterized from a biochemical and genetic point of view and has served as a model system. Nevertheless, a molecular understanding of the details of *E. coli* transcription and its regulation, and therefore its full exploitation as a model system, has been hampered by the absence of high-resolution structural information on *E. coli* RNA polymerase (RNAP). We use a combination of approaches, including high-resolution X-ray crystallography, ab initio structural prediction, homology modeling, and single-particle cryo-electron microscopy, to generate complete atomic models of *E. coli* core RNAP and an *E. coli* RNAP ternary elongation complex. The detailed and comprehensive structural descriptions can be used to help interpret previous biochemical and genetic data in a new light and provide a structural framework for designing experiments to understand the function of the *E. coli* lineage-specific insertions and their role in the *E. coli* transcription program.

## Abstract 2

A combination of structural approaches yields a complete atomic model of the highly biochemically characterized *Escherichia coli* RNA polymerase, enabling fuller exploitation of *E. coli* as a model for understanding transcription.

## Author Summary

Transcription, or the synthesis of RNA from DNA, is one of the most important processes in the cell. The central enzyme of transcription is the DNA-dependent RNA polymerase (RNAP), a large, macromolecular assembly consisting of at least five subunits. Historically, much of our fundamental information on the process of transcription has come from genetic and biochemical studies of RNAP from the model bacterium *Escherichia coli.* More recently, major breakthroughs in our understanding of the mechanism of action of RNAP have come from high resolution crystal structures of various bacterial, archaebacterial, and eukaryotic enzymes. However, all of our high-resolution bacterial RNAP structures are of enzymes from the thermophiles *Thermus aquaticus* or *T. thermophilus*, organisms with poorly characterized transcription systems. It has thus far proven impossible to obtain a high-resolution structure of *E. coli* RNAP, which has made it difficult to relate the large collection of genetic and biochemical data on RNAP function directly to the available structural information. Here, we used a combination of approaches—high-resolution X-ray crystallography of *E. coli* RNAP fragments, ab initio structure prediction, homology modeling, and single-particle cryo-electron microscopy—to generate complete atomic models of *E. coli* RNAP. Our detailed and comprehensive structural models provide the heretofore missing structural framework for understanding the function of the highly characterized *E. coli* RNAP.

## Introduction

**Figure 1:**
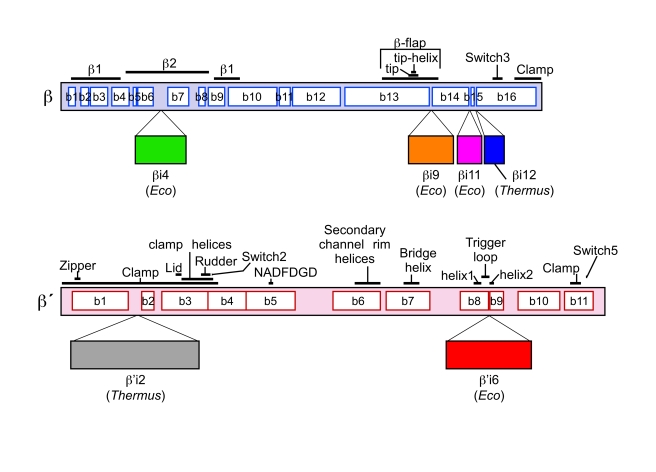
Sequence architecture of the bacterial RNAP large subunits. The vertical bars represent the primary sequence of the bacterial RNAP β (top, light cyan) and β' (bottom, light pink) subunits.The white boxes indicate sequence regions common to all bacterial RNAPs, as defined by Lane et al. [3]. Important structural features are labeled above the bars [19]. Lineage-specific insertions (labeled according to the nomenclature of Lane et al. [3] are shown below the bars. The color-coding for the large subunits and the lineage-specific insertions shown here is used throughout this article.

RNA in all cellular organisms is synthesized by a complex molecular machine, the DNA-dependent RNA polymerase (RNAP). In bacteria, the catalytically competent core RNAP (subunit composition α_2_ββ'ω) has a molecular mass of ∼400 kDa. Evolutionary relationships for each of the bacterial core subunits have been identified between all organisms from bacteria to man [1]–[3]. These relationships are particularly strong between the two largest subunits, β' and β, which contain colinearly arranged segments of conserved sequence (Figure 1) [3]. These conserved segments are separated by relatively nonconserved spacer regions in which large, lineage-specific gaps or insertions can occur [3],[4]. The functional significance of these lineage-specific differences is poorly understood due to a lack of correlated biochemical and structural information. The bulk of our biochemical and genetic knowledge on bacterial RNAP comes from studies of *Escherichia coli* (*Eco*) RNAP but all of our high-resolution structural information comes form *Thermus* RNAPs [5]–[8] as *Eco* RNAP has not been amenable to X-ray crystallography analysis. The *Eco* and *Thermus* β and β' subunits harbor large sequence insertions (>40 amino acids) that are not present in the other species and are not shared across bacterial species (Figure 1) [3]. For example, the *Eco* β' subunit contains β'-insert-6 (or β'i6, using the lineage-specific insert nomenclature of Lane et al. [3]), a 188-residue insertion in the middle of the highly conserved “trigger loop.” On the other hand, the *Thermus* β' subunit lacks β'i6 but contains β'i2 (283 residues). High-resolution structures of both of these lineage-specific inserts reveal that they comprise repeats of a previously characterized fold, the sandwich-barrel hybrid motif (SBHM) [9],[10]. Similarly, the *Eco* β subunit harbors three large insertions missing in *Thermus*, βi4 (119 residues), βi9 (99 residues), and βi11 (54 residues), whereas the *Thermus* β subunit harbors βi12 (43 residues).

In some respects, the high-resolution *Thermus* RNAP structures have served as good models to interpret the functional literature obtained from biochemical, biophysical, and genetic studies of *Eco* RNAP [11],[12]. Nevertheless, a complete molecular model of *Eco* core RNAP has not been available due to the absence of high-resolution structural information on the *Eco* β subunit lineage-specific inserts. The most detailed structural studies of *Eco* RNAP have come from cryo-electron microscopy (cryo-EM) analysis of helical crystals at about 15 Å-resolution [13]. This cryo-EM reconstruction of *Eco* core RNAP could be interpreted in detail by fitting the *Taq* core RNAP X-ray structure, revealing a large distortion of the structure (opening of the active site channel by more than 20 Å) due to intermolecular contacts in the helical crystals. Strong electron density for *Eco* βi9 was present in the cryo-EM reconstruction, but weak density for *Eco* βi4 and *Eco* β'i6 indicated these domains were flexible in the context of the helical crystals [13]. Most previous EM reconstructions of various forms of *Eco* RNAP have not revealed information concerning the lineage-specific inserts (for instance, see [14]). A recent 20 Å-resolution, negative-stain EM reconstruction of an activator-dependent transcription initiation complex containing *Eco* RNAP [15] allowed the positioning of the *Eco* β'i6 crystal structure [10], but the lack of structural information on the other *Eco* lineage-specific inserts prevented the detailed interpretation of additional densities present in the reconstruction [15].

In this study, we used a combination of structural approaches to generate a complete molecular model of *Eco* core RNAP. We determined two new high-resolution X-ray crystal structures of *Eco* RNAP β subunit fragments that include *Eco* βi4 and βi9 and used an ab initio method to predict the structure of the small *Eco* βi11 [16]. The three available X-ray crystal structures of *Eco* RNAP fragments (the two structures determined herein and the structure of *Eco* β'i6 [10]) and the predicted structure of *Eco* βi11 were incorporated into a homology model of *Eco* core RNAP. Finally, we used cryo-EM imaging combined with single-particle image analysis to obtain a low-resolution structure of the solution conformation of *Eco* core RNAP in which densities corresponding to lineage-specific insertions could be clearly identified. Flexible-fitting of the *Eco* RNAP homology model into cryo-EM densities generated a complete molecular model of *Eco* core RNAP and an *Eco* RNAP ternary elongation complex (TEC).

## Results

### Crystal Structure of *Eco* RNAP β2-βi4

**Figure 2:**
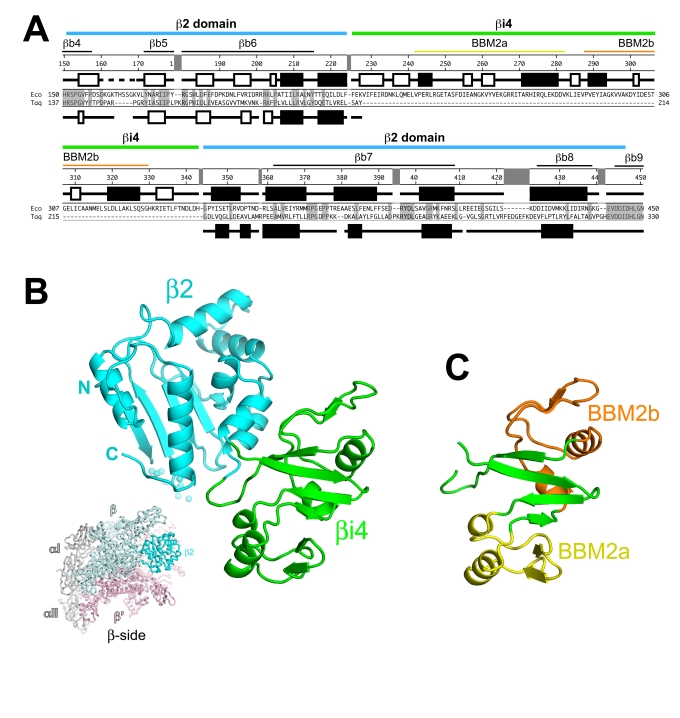
Sequence and structure of *Eco* RNAP β2-βi4. (A) Sequence alignment comparing *Eco* RNAP β2-βi4 with the corresponding region of *Taq* (which lacks βi4). Shaded residues are identical between the two sequences. The secondary structures are indicated directly above (for *Eco*) and below (for *Taq*) the sequences; filled rectangles denote α-helices, open rectangles denote β-strands, the dashed lines denote disordered regions. The number scale above the *Eco* secondary structure corresponds to the *Eco* β subunit sequence. Above the number scale, black lines denote the sequence regions common to all bacterial RNAPs [3]. The yellow and orange lines denote the two BBM2 motifs [4]. The extent of the common β2 domain (thick cyan line) and the lineage-specific insert βi4 (thick green line) is indicated at the top. (B) Ribbon diagram of *Eco* β2-βi4 (β2 domain, cyan; βi4, green). A disordered loop (*Eco* β 161–169) is denoted by small spheres. The view corresponds to the reference view of *Taq* core RNAP (lower left, β-side view), shown as a backbone worm and color-coded as follows: αI, αII, ω, gray; β', light pink; β, light cyan, except the β2 domain is colored cyan and labeled. (C) Ribbon diagram of *Eco* βi4 (same view as B). The tandem BBM2 motifs predicted by Iyer et al. [4] are color-coded as in (A) (BBM2a, yellow; BBM2b, orange).

The lineage-specific insert βi4 (previously named β dispensable region 1, or βDR1, or SI1 in the literature [13],[17],[18]), located between bacterial shared regions βb6 and βb7 (using the bacterial RNAP common region nomenclature of Lane et al. [3]) in the β2 domain (Figure 1) [5],[19], was predicted to comprise from one to six tandem repeats of a structural motif termed the β-β' module 2 (BBM2) [4]. The βi4 of Acidobacteria, Mollicutes, and Proteobacteria (including *Eco*) was predicted to comprise two tandem BBM2 repeats [3]. *Eco* βi4 comprises β residues 225–343 (Figure 2A).

We prepared a construct comprising the *Eco* β2 domain including βi4 inserted within it (*Eco* β residues 152–443, hereafter called *Eco* β2-βi4). After reductive methylation [20], the protein formed crystals that diffracted X-rays to 1.6 Å-resolution (Table 1). The structure was solved by single-anomalous dispersion using a dataset collected from crystals of selenomethionyl-substituted protein [21] and refined to an *R*/*R*_free_ of 0.209/0.229 at 1.6 Å-resolution (Table 1, Figures 2, S1).

**Table 1:** Crystallographic statistics for *Eco* RNAP β2-βi4 crystals.

	Se1^a^	Se2
**Data collection**		
Space group	P2_1_2_1_2	P2_1_2_1_2
Cell dimensions		
*a*, *b*, *c* (Å)	106.28, 51.84, 61.77	106.31, 52.04, 61.83
α β γ (°)	90, 90, 90	90, 90, 90
	*Peak*	*Remote*
Wavelength	0.9785	0.9919
Resolution (Å)	25.0–1.90 (1.97–1.90)	25.0–1.60 (1.64–1.60)
*R*_sym_	0.081 (0.596)	0.0690 (0.416)
*I*/σ*I*	11.0 (2.7)	40 (5.1)
Completeness (%)	94.1 (87.1)	98.5 (94.0)
Redundancy	2.6 (2.4)	7.0 (6.5)
**Refinement**		
Resolution (Å)		25.0–1.60
No. reflections		42,737
*R*_work_/*R*_free_		0.209/0.229
No. atoms		
Protein		2,345
Water		386
*B*-factors		
Protein		14.51
Water		24.58
R.m.s deviations		
Bond lengths (Å)		0.008
Bond angles (°)		1.134

a Scaling statistics for Se1 dataset calculated without combining anomalous pairs.

As expected, the *Eco* β2 (*Eco* β residues 151–224 and 344–445) and the *Thermus* β2 (*Taq* or *Tth* β residues 138–325) domains have similar overall structures (Figure S2). A superimposition of the two domains over 100 residues (excluding flexible loops connecting secondary structural elements) yields a root-mean-square deviation in α-carbon positions of 1.68 Å. Significant differences in the structures include: (i) the loop connecting the first two β-strands of the β2 domain, where *Eco* has a 5-residue insertion (*Eco* β residues 164–168, disordered in our structure), and (ii) the loop connecting the last two α-helices of the β2 domain, which includes a 7-residue insertion present in *Taq* β (*Taq* β residues 293–299; Figures 2A, S2).

The βi4 domain is inserted at the surface of the β2 domain distal to the connection with the RNAP (Figure 2B). A 3-residue segment of *Taq* β (*Taq* β 212–214) is replaced by the 119-residue *Eco* βi4 (Figure 2A). The *Eco* βi4 folds into a compact, cylinder-shaped domain about 22 Å in diameter and about 50 Å in length (Figures 2B, 2C). The compact domain is connected to the β2 domain by two short connector loops (*Eco* β 225–226 and 337–345). The βi4 domain packs against β2, resulting in the burial of a modest 618 Å^2^ of surface area. As predicted [4], the *Eco* βi4 includes two tandem BBM2 motifs (Figure 2A, 2C).

### Crystal Structure of *Eco* RNAP βflap-βi9

**Figure 3:**
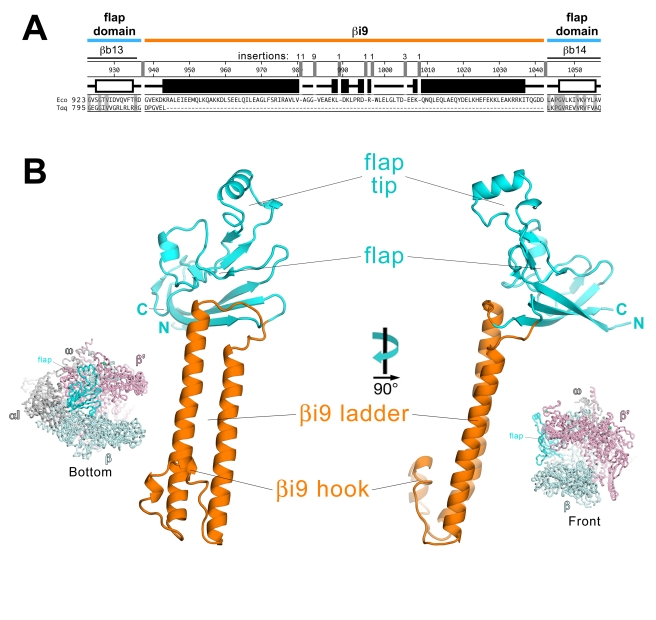
Sequence and structure of *Eco* RNAP βflap-βi9. (A) Sequence alignment comparing the sequence context of *Eco* RNAP βi9 with the corresponding region of *Taq* (which lacks βi9). Shaded residues are identical between the two sequences. The secondary structure for *Eco* is indicated directly above the sequence; filled rectangles denote α-helices, open rectangles denote β-strands. The number scale above the *Eco* secondary structure corresponds to the *Eco* β subunit sequence. Above the number scale, black lines denote the sequence regions common to all bacterial RNAPs [3]. Gaps in the βi9 sequence with numbers above denote the location and residue length of insertions in an alignment of 307 non-redundant βi9 sequences (see Supporting Information). The extent of the common βflap domain (thick cyan line) and the lineage-specific insert βi9 (thick orange line) is indicated at the top. (B) Two orthogonal views of *Eco* βflap-βi9 (βflap, cyan; βi9, orange). The views correspond to the reference views of *Taq* core RNAP (left, bottom view; right, front view), shown as a backbone worm and color-coded as follows: αI, αII, ω, gray; β', light pink; β, light cyan, except the βflap domain is colored cyan and labeled.

The lineage-specific insert βi9 (previously named β dispensable region 2, or βDR2, or SI2 in the literature [13],[18],[22],[23]) is located between bacterial shared regions βb13 and βb14 [3] at the base of the flap domain (Figure 1) [5],[19]. The βi9 is found in Acidobacteria, Aquificae, Bacteriodetes, Chlamydiae, Chlorobi, Planctomycetes, Proteobacteria (including *Eco*), and Nitrospirae [3]. *Eco* βi9 comprises β residues 938–1042 (Figure 3A).

A construct comprising the *Eco* flap domain (*Eco* β 831–1057), including βi9, was crystallized as a complex with bacteriophage T4 gp33 (K.-A.F.T., P. Deighan, S. Nechaev, A. Hochschild, E.P. Geiduschek, S.A.D., in preparation). The structure was solved by a combination of molecular replacement (using the *Taq* flap domain as a search model) and single-anomalous dispersion using data collected from selenomethionyl-substituted protein (Table S1, Figure S3) [21]. The complete structure was refined to an *R*/*R*_free_ of 0.264/0.291 at 3.0 Å-resolution. T4 gp33 interacts primarily with the flap-tip and does not make any interactions with βi9. These and further details of the complex with T4 gp33 will be described elsewhere (K.-A.F.T., P. Deighan, S. Nechaev, A. Hochschild, E.P. Geiduschek, S.A.D., in preparation).

The βi9 domain is inserted at the base of the flap domain, near the C-terminal connection of the flap with the rest of the RNAP and distal to the flap-tip (Figure 3B). A 6-residue segment of *Taq* β (*Taq* β 809–814) is replaced by the 105-residue *Eco* βi9 (Figure 3A). The *Eco* βi9 comprises two long, parallel α-helices of 38 and 32 residues (*Eco* β 943–980 and 1006–1037, respectively) with a short, hook-like connecting segment (residues 981–1005) at the end distal to the flap (Figure 3B), forming an apparently rigid structure reminiscent of a hook-and-ladder that extends nearly 65 Å out from the flap domain. The βi9 is connected to the flap domain by two connector loops (*Eco* β 938–942 and 1038–142) but makes minimal interactions with the flap itself. The structure does not appear to conform to the β-β' module 1 motif (BBM1, similar to the BBM2 motif, Figure 2C) predicted for βi9 [4]. The 105-residue *Eco* βi9 is at the lower end of the size range for βi9 sequences, which ranges from 105 residues in some Proteobacteria to 143 residues in some Bacteriodetes. An alignment of 307 non-redundant βi9 sequences (see Dataset S1) reveals that the two long, ladder α-helices do not harbor insertions; all of the insertions occur in the hook-like connector at the distal end of βi9 (Figure 3A). Therefore, we conclude that βi9 has a conserved core structure with the two ladder α-helices of conserved length.

### Cryo-EM Reconstruction of *Eco* RNAP

**Figure 4:**
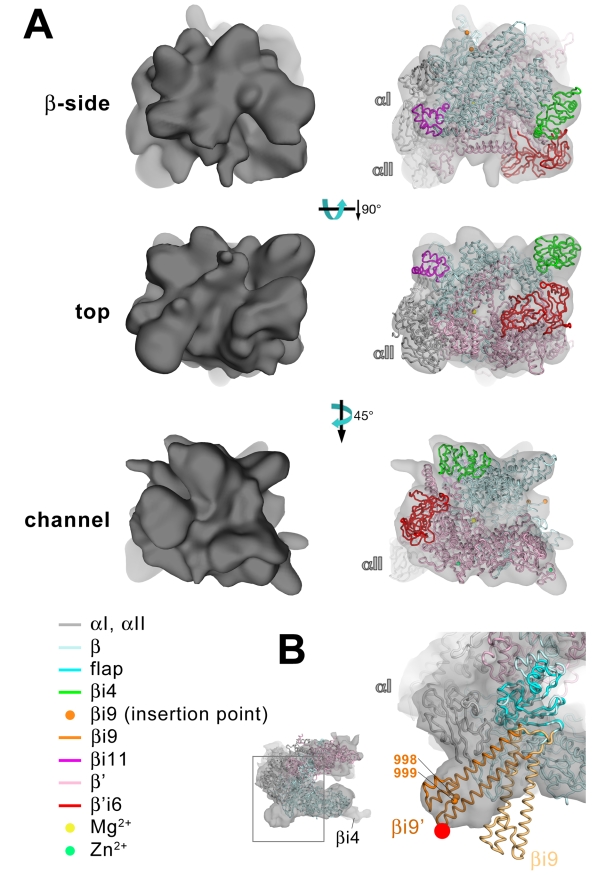
Fitting into cryo-EM densities to generate a molecular model of *Eco* RNAP. (A) Three views of the spEM density map and corresponding fit of the *Eco* RNAP homology model (excluding ω, the C-terminal 41 residues of β', and βi9). For each view (β-side, top, and channel views), the left image shows the spEM density map (grey surface, contoured at 2.5 σ), and the right image shows the spEM density map (grey transparent surface) with the fitted *Eco* RNAP homology model superimposed. The *Eco* RNAP homology model is shown as a backbone worm, color-coded as shown in the key (lower left). (B) View of the hEM density map and corresponding fit of the *Taq* core RNAP crystal structure [13]. The small view (left, which corresponds roughly to the bottom view) shows the entire structure (weak density due to βi4 is noted). The boxed region is magnified on the right, where the *Eco* βflap-βi9 structure (βflap, cyan; βi9, light orange) is superimposed via the flap domain (excluding the flap-tip). The resulting position of βi9 (light orange) was adjusted to fit into the hEM density (βi9', orange). The red dot denotes the position of a positive difference peak from a hEM reconstruction of a mutant RNAP harboring a 234-residue insertion in βi9 between residues 998 and 999 [23].

We generated a single-particle cryo-EM (spEM) reconstruction of *Eco* RNAP by analyzing ∼42,000 images of *Eco* RNAP particles preserved in vitreous ice (Figures 4A, S4–S6). Initial image orientation parameters were determined using a 35 Å-resolution RNAP model based on the *Taq* core RNAP X-ray structure [5]. Final refinement of image orientation parameters by projection matching yielded a structure of *Eco* RNAP with a 0.5 Fourier-shell cutoff resolution of ∼11.2 Å (Figure S4). Nevertheless, information beyond about 14 Å resolution was very weak, and so the figures and analysis described herein were performed on a low-pass Fourier-filtered map [24],[25]. Although the cryo-EM grids were prepared with samples of *Eco* RNAP holoenzyme (core RNAP plus the promoter-specificity σ^70^ subunit), the σ^70^ subunit apparently dissociated during grid preparation as density corresponding to σ^70^ was completely absent. Dissociation during cryo-EM sample preparation has been noted for other macromolecular complexes [26] and is also consistent with reports of dissociation constants for the σ^70^/core RNAP complex as high as 200–300 nM (the RNAP concentration used here was about 200 nM). The spEM reconstruction showed *Eco* core RNAP in a conformation similar to that observed in *Thermus* X-ray structures but with clear density corresponding to βi4, βi11, and β'i6 (Figures 4A, S5, S6).

### Molecular Model of the Complete *Eco* Core RNAP

In order to interpret the spEM map of *Eco* core RNAP, we generated a homology model of *Eco* core RNAP using the core component of the *T. thermophilus* (*Tth*) RNAP holoenzyme structure (PDB ID 1IW7) [7] as a template. The locations of the *Eco* lineage-specific insertions βi4, βi9, βi11, and β'i6 (absent in *Thermus*) were left as gaps in the *Eco* sequences. *Thermus*-specific inserts βi12 and β'i2 (Figure 1) were also removed from the structural template. The crystal structures of *Eco* β2-βi4 (Figure 2B) and βflap-βi9 (Figure 3B) were spliced into the resulting homology model by superimposition of the overlapping β2 and βflap domains, respectively. At this stage, the *Eco* RNAP model was readily fit manually into the spEM map. The spEM map contained clear density corresponding to βi4, but density for βi9 was absent. Density for the ω subunit as well as the C-terminal helix of β' were also absent. In addition, extra density not accounted for by the homology model was present for βi11 and β'i6. An ab initio predicted structure of the short βi11 (see below) was placed into the corresponding density to fill in the gap in the *Eco* β sequence between 1121 and 1181. The crystal structure of *Eco* β'i6 (PDB ID 2AUK) [10] was readily fit manually into excess density in the vicinity of its insertion point in β'. Two criteria were used to determine the orientation of β'i6 with respect to the rest of the RNAP. First, although β'i6 comprises a tandem repeat of two SBHM domains, the C-terminal SBHM domain (SBHMb) [10] harbors larger insertions between the core SBHM β-strands, making β'i6 asymmetric in shape. The asymmetry is clearly seen in the spEM density as well (see Figure 4A, top view). Moreover, only one orientation of β'i6 allows connection to the gap in the *Eco* β' sequence (between residues 940 and 1132) without severe distortion. The positioned β'i6 was readily connected to the open (unfolded) trigger-loop (TL) conformation of the model.

Flexible-fitting of the final *Eco* RNAP model (excluding ω, the C-terminal 41 residues of β', and βi9) into the spEM map was performed using YUP.SCX [27], resulting in a superb fit of the conserved RNAP as well as of the lineage-specific inserts (excluding βi9; Figures 4A, S5, S6). In order to position βi9 in the context of the entire RNAP structure, we used our previously determined helical cryo-EM map of *Eco* core RNAP (hEM) and fit of the *Taq* core RNAP X-ray crystal structure [13] since the hEM map contains strong density for βi9. The βflap portion (excluding the flexible flap-tip) of the *Eco* βflap-βi9 crystal structure (Figure 3B) was superimposed on the *Taq* βflap domain in the context of the *Taq* RNAP fit into the hEM density. The resulting position of βi9 did not correspond to the hEM density (light orange, βi9 in Figure 4B) but was fit into the density by a rotation of about 35° (orange, βi9' in Figure 4B). This positioning of βi9 is consistent with the location of positive difference density observed in the context of the helical crystals due to a 234-residue insertion between *Eco* β residues 998 and 999 (red dot, Figure 4B). The *Eco* core RNAP model was completed by adding back the C-terminal segment of β' as well as ω (in accordance with the *Thermus* RNAP structures).

The *Eco* core RNAP model was then used as the basis for generating a homology model of an *Eco* TEC, using the *Tth* TEC crystal structure (open TL conformation, PDB ID 2O5I) [8]. For both models, the lineage-specific inserts (βi4, βi9, βi11, β'i6 for *Eco*; β'i2 and β'i12 for *Tth*) were removed. The nucleic acids present in the *Tth* crystal structure were fixed during the modeling. The *Eco* lineage-specific inserts were added back to the resulting TEC model (according to their positions in the *Eco* core RNAP model), and missing portions of the nucleic acids (the upstream double-stranded DNA, and the nontemplate strand of the DNA within the transcription bubble) were modeled according to Korzheva et al. [28].

## Discussion

In this work, two new X-ray crystal structures (*Eco* β2-βi4, Figure 2; *Eco* βflap-βi9, Figure 3) and an ab initio predicted structure (*Eco* βi11, see below), combined with a previously determined X-ray crystal structure of *Eco* β'i6 [10], provide high-resolution structural descriptions of each of the lineage-specific sequence insertions found in the highly biochemically and genetically characterized *Eco* RNAP [3]. In addition, a new 15 Å-resolution cryo-EM single-particle reconstruction of *Eco* RNAP (Figures 4A, S4–S6) reveals clear electron density for βi4, βi11, and β'i6, while a previously determined cryo-EM reconstruction of *Eco* core RNAP from helical crystals contains strong electron density for βi9 [13],[23]. The combination of these structural data provides the basis for a detailed and complete atomic model of *Eco* RNAP and an *Eco* core RNAP TEC.

The large β and β' subunits comprise regions of sequence shared among all bacterial RNAPs [3]. These shared regions, which make up 63% of the *Eco* β and 67% of the *Eco* β' sequence, are expected to have nearly identical structure among all bacterial RNAPs. The α subunits are also highly homologous [5],[29]. Thus, most of the *Eco* RNAP structure is expected to be highly similar, if not identical, to the *Thermus* RNAP structures. The unique contribution of this work is the high-resolution structural information on the *Eco* lineage-specific inserts βi4, βi9, and βi11, as well as the detailed structural model of all the lineage-specific inserts in the context of the entire RNAP and a TEC. The following discussion therefore focuses on the *Eco* lineage-specific inserts and insights into their role in RNAP function provided by our new structural information.

### βi4

RNAPs harboring deletions or insertions within βi4 support cell growth and retain basic in vitro transcription function, leading to its designation as “dispensable region I” of the β subunit [17]. Nevertheless, careful studies of a nearly precise βi4 deletion (deletion of *Eco* β 226–350) revealed defects [18]. The purified Δβi4-RNAP showed only very mild defects, or no defects at all, in a number of in vitro tests [17],[18]. In vivo, however, the Δβi4-RNAP was unable to support cell growth at 42°C and could only support slow growth at 30°C.

**Figure 5:**
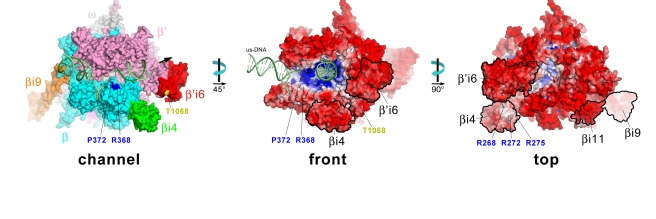
Three views (channel, front, and top) of the *Eco* RNAP TEC model. In each view, the RNAP is shown as a molecular surface, and the nucleic acids are shown as phosphate backbone worms (DNA template strand, dark green; DNA nontemplate strand, light green; RNA transcript, gold). Channel view (left): The RNAP is color coded as follows: αI, αII, ω, grey; β, cyan, except βi4 is green, βi9 is orange, and βi11 is magenta; β', pink, except β'i6 is red. The positions of two *paf* mutants (βR368 and βP372) [17],[32] are colored blue. β'T1068 (within β'i6), which is phosphorylated by bacteriophage T7 Gp0.7 [48], is shown in yellow. The thick black arrow points in the downstream direction. Front view (middle): The RNAP molecular surface is colored according to the solvent-exposed electrostatic surface distribution [67], scaled from –10 kT (red) to +10 kT (blue). The locations of the *paf* mutants βR368 and βP372, and β'T1068, are denoted. The upstream DNA (us-DNA) is labeled. Top view (right): The RNAP molecular surface is colored according to the solvent-exposed electrostatic surface distribution [67], scaled from −10 kT (red) to +10 kT (blue). The locations of highly conserved basic residues in βi4 (βR268, R272, and R275) are denoted. In this view, the nucleic acids are fortuitously hidden from view.

In our model of the *Eco* TEC, βi4 extends out from the β2 domain roughly in the direction of the downstream double-stranded DNA (Figure 5). However, βi4 is unlikely to interact directly with the downstream DNA to form part of an extended DNA binding channel since βi4 tilts away from the DNA, creating a roughly 15 Å gap between itself and the DNA. Moreover, the solvent-exposed surface of βi4, including the entire surface facing the DNA, is highly acidic (Figure 5, front view), except for a “neutral patch” that arises from three conserved residues, *Eco* β R268, R272, and R275 (Figure 5, top view). These positions are conserved as basic residues (either R or K) in 98%, 91%, and 91% of the sequences, respectively, in an alignment of 316 non-redundant βi4 sequences (containing only “*Eco*-like” βi4 sequences comprising two BBM2 domains; see Dataset S2) and may comprise an interaction determinant for an as yet unidentified regulatory factor.

The bacteriophage T4 Alc protein interacts with the host *Eco* RNAP [30] and causes premature transcription termination on *Eco* DNA while allowing *Eco* RNAP-mediated transcription of phage DNA containing 5-hydroxymethylcytosine [31]. *Eco paf* mutants (prevent Alc function) have been mapped to the *rpoB* gene encoding the RNAP β subunit [17],[32]. *Eco* β mutants R368H, R368C, and a double mutant (P345S/P372L) display the *paf* phenotype, possibly by directly preventing Alc interaction with RNAP [17]. These mutations lie within a region of the β subunit that could be deleted without disrupting basic transcription function [17] but are not, in fact, contained within βi4 (Figure 2A). Two of the mutated positions (368 and 372) lie within βb7, a region shared among all bacterial RNAPs (Figure 2A) [3]. In our structural model of the *Eco* RNAP TEC, βR368 and βP372 lie within a structural feature that sits at the entrance of the main RNAP active site channel, inside the “V” formed by the upstream and downstream DNA of the TEC (Figure 5, channel and front views). These residues are not near any nucleic acids in the TEC (the closest approach is for the backbone carbonyl of βP372, which is 15 Å away from the nontemplate DNA phosphate backbone at the -10 position) but could comprise part of an Alc binding determinant on the RNAP [17]. The 19 kDa Alc protein bound in this vicinity (Figure 5, channel and front views) would be well positioned to distinguish the presence of cytosine or 5-hydroxymethylcytosine in either the downstream double-stranded DNA (where the 5-hydroxymethyl moiety would be exposed in the major groove) or the single-stranded non-template DNA in the transcription bubble.

### βi9

RNAPs harboring deletions or insertions within βi9 support cell growth and retain in vitro transcription function, leading to its designation as “dispensable region II” of the β subunit [17],[22],[23],[33]. Nevertheless, careful studies of a precise βi9 deletion (deletion of *Eco* β 938–1040) revealed defects [18]. The purified Δβi9-RNAP showed only very mild defects, or no defects at all, in a number of in vitro tests [18]. The βi9 contains the epitope for the PYN-6 monoclonal antibody and, consistent with in vitro tests showing little effect of deleting βi9 on normal RNAP function, RNAP can be immobilized using the PYN-6 antibody but remains active for in vitro transcription [22]. In vivo, however, the Δβi9-RNAP was unable to support cell growth in minimal media [18].

**Figure 6:**
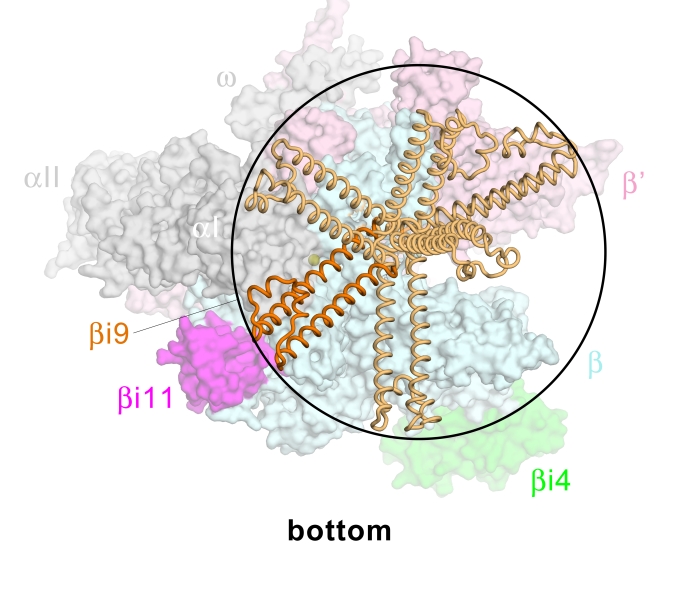
Orientational flexibility of βi9. Bottom view of the *Eco* RNAP model. The RNAP is shown as a molecular surface (αI, αII, ω, grey; β, light cyan, except βi4 is green and βi11 is magenta; β', light pink) except for βi9, which is shown as a backbone worm. The modeled position of βi9 (see Figure 4B) is colored orange. Selected alternative orientations accessible to βi9 are colored light orange. The potential reach of βi9 maps out roughly a hemisphere with a radius of 65 Å.

Our crystal structure of the *Eco* βflap-βi9 suggests that βi9 is attached to the flap via flexible linkers and does not make a significant, stable interaction with the flap (Figure 3B), suggesting that βi9 is highly flexible in its orientation with respect to the flap. Indeed, the position of βi9 in the βflap-βi9 crystal structure appears to be determined by packing interactions with neighboring, symmetry-related molecules. In keeping with this, there is no density for βi9 in the spEM reconstruction (Figures 4A, S5, S6). However, in our previous hEM reconstruction of *Eco* RNAP, strong density consistent with βi9 was observed, and this density was shown to correspond to βi9 through a helical reconstruction of a mutant RNAP harboring a large insertion between positions 998 and 999 [23]. In the helical crystals, the packing of a neighboring, symmetry-related RNAP molecule restricts the range of positions available to βi9, allowing its visualization (Figure 4B). Fitting βi9 into the corresponding density in the hEM reconstruction required a large change in the position of βi9 with respect to the flap, but the final model fits very well into the density and is also consistent with the EM localization results [23], which were not used as a constraint in the fitting (Figure 4B). This model for the position of βi9 in the context of the entire RNAP is presented as an example of a particular orientation that is possible for βi9 (since it was observed in the helical crystals), but the evidence indicates that βi9 does not adopt a particular conformation with respect to the RNAP but can access a wide range of positions (Figure 6).

The modeled position of βi9 is not near any nucleic acids in the TEC or in the open promoter complex [34]. Moreover, the solvent-exposed surface of βi9 is primarily acidic (Figure S7). Interestingly, an alignment of 307 non-redundant βi9 sequences (see Dataset S1) reveals that conserved, solvent-exposed residues are all displayed on the back face of the “ladder,” opposite the “hook” (Figure S7). Conserved features of this face comprise charged residues D959 (conserved as D or E in 97% of the sequences), E962 (D/E, 95%), R974 (K/R, 89%), K1032 (K/R, 95%), and K1035 (K/R, 94%), and one conserved hydrophobic residue, I966. These features suggest that this face of the ladder may serve as an interaction determinant for as yet unidentified regulatory factors. D959 and K1032 participate in an apparently conserved salt bridge. Predictably, a number of conserved hydrophobic residues participate in the hydrophobic core of the domain, either between the ladder and the hook (L979, L989) or in the packing interface between the two ladder helices (L1029, I1036).

### βi11

**Figure 7:**
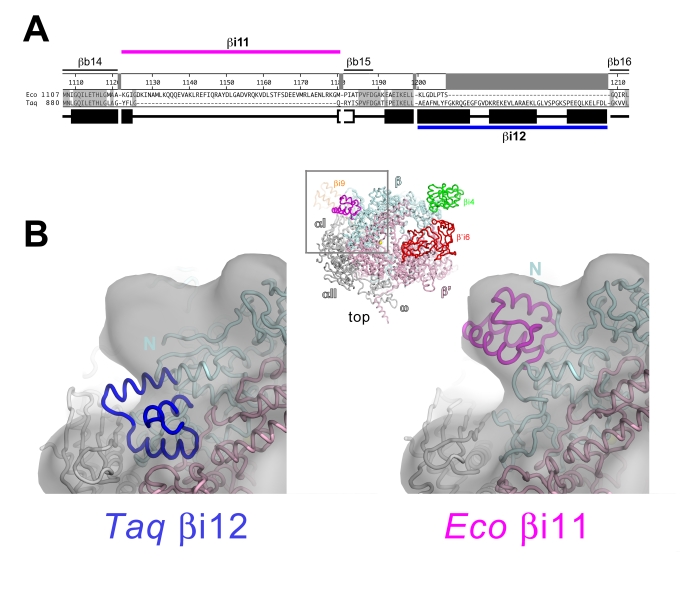
Sequence and structural context of *Eco* RNAP βi11 and *Taq* βi12. (A) Sequence alignment comparing the sequence context of *Eco* RNAP βi11 with the corresponding region of *Taq* (which lacks βi11 but harbors βi12) [3]. Shaded residues are identical between the two sequences. The experimentally determined secondary structure for *Taq* is indicated directly below the sequence; filled rectangles denote α-helices, open rectangles denote β-strands. The number scale above the *Eco* secondary structure corresponds to the *Eco* β subunit sequence. Above the number scale, black lines denote the sequence regions common to all bacterial RNAPs [3]. The extent of *Eco* βi11 and *Taq* βi12 are denoted by the thick magenta line (above) and the thick blue line (below). (B) A portion of the spEM map (contoured at 2.5 σ) is shown (transparent grey surface) with the superimposed *Taq* core RNAP structure (left, with βi12 colored blue) and the fitted *Eco* RNAP model (right, with βi11 colored magenta). The view corresponds roughly to the reference view of the *Eco* RNAP model (top view), shown as a backbone worm and color-coded as follows: αI, αII, ω, gray; β', light pink, except β'i6 is red; β, light cyan, except βi4 is green, βi9 is orange, and βi11 is magenta.

The lineage-specific insert βi11 is located between bacterial shared regions βb14 and βb15 (Figures 1, 7A) [3]. The βi11 is found in Acidobacteriaceae, Aquificae, and Proteobacteria (including *Eco*) [3]. In each bacterial species where it is found, βi11 has a length ranging from 54–69 residues. Comparing *Taq* with *Eco*, a 5-residue segment of *Taq* β (*Taq* β 895–899) is replaced by the 59-residue *Eco* βi11, comprising *Eco* β residues 1122–1180 (Figure 7A).

Although a construct corresponding to *Eco* RNAP βi11 overexpressed and was well behaved, we were unable to obtain crystals suitable for X-ray analysis. The Robetta server (http://robetta.bakerlab.org/) provided an ab initio predicted structure of this short, 59-residue fragment (Figure S8) that is consistent with a number of observations from our structural and sequence analyses:

- The overall predicted structure of βi11 fits well into the corresponding spEM density (Figure 7B, right).
- The termini of the predicted βi11 structure could be readily connected to the corresponding gap in the *Eco* RNAP β structure with only minor modifications.
- In an alignment of 310 non-redundant βi11 sequences (see Dataset S3), insertions and gaps occur in locations consistent with the predicted structure (i.e. in loops connecting secondary structural elements and away from the RNAP; Figure S8).
- Analysis of the βi11 sequence alignment reveals that most of the conserved residues are hydrophobic in nature and are buried in the hydrophobic core of the βi11 fold (Figure S8C). Two conserved, solvent-accessible polar residues (R1142 and D1166) form an apparently conserved salt-bridge that may stabilize the structure (Figure S8C).

The βi11 was only recently recognized as a distinct, lineage-specific insertion [3],[4]. To our knowledge, no information on the effects of deletions or mutations in this region is available. Inspection of the spEM map and the aligned X-ray structure of *Taq* core RNAP in the region of the β subunit between shared regions βb14 and βb16 revealed a clear discrepancy that corresponds to *Taq* βi12 (Figure 7B). In our *Eco* RNAP model, the *Taq* βi12 was removed and the resulting gap was connected by the loop corresponding to *Eco* β residues 1200–1207. The predicted structure of *Eco* βi11 (Figure S8) was then spliced between *Eco* β residues 1121 and 1181 and oriented to fit into the EM density, resulting in a good fit. The resulting location of *Eco* βi11 clashed with the position of the β-subunit N-terminus, which was redirected to relieve the clash (Figure 7B).

### β'i6

While the large *Eco* lineage-specific insertions βi4 and βi9 appear to play only peripheral roles in RNAP function, and the complete deletion of either one results in relatively minor growth defects [18], β'i6 plays a more important role in *Eco* RNAP function. Complete deletion, or even partial deletion, of β'i6 is not viable [18],[35]. Complete deletion causes a severe defect in RNAP assembly, both in vivo and in vitro [18],[35], but the in vivo–assembled Δβ'i6-RNAP can be obtained from cells simultaneously overexpressing the other RNAP subunits [18], and partial deletions of β'i6 can be assembled in vitro [35]. Biochemical studies of enzymes with complete or partial β'i6 deletions reveal a number of severe defects. The Δβ'i6-RNAP forms dramatically destabilized open promoter complexes [18]. RNAPs harboring partial deletions in β'i6 are defective in transcript cleavage and have a dramatically reduced transcript elongation rate at subsaturating NTP concentrations [35]. Antibody binding to epitopes within β'i6 inhibit transcription as well as intrinsic transcript cleavage [35],[36].

The β'i6 plays a central role in the pausing/termination behavior of elongating *Eco* RNAP [18],[35]. Full or partial deletions in β'i6 result in RNAPs with dramatically altered pausing behavior [18],[35]. A genetic screen for termination-altering mutants in *Eco* RNAP uncovered 10 positions scattered throughout β'i6 [37].

**Figure 8:**
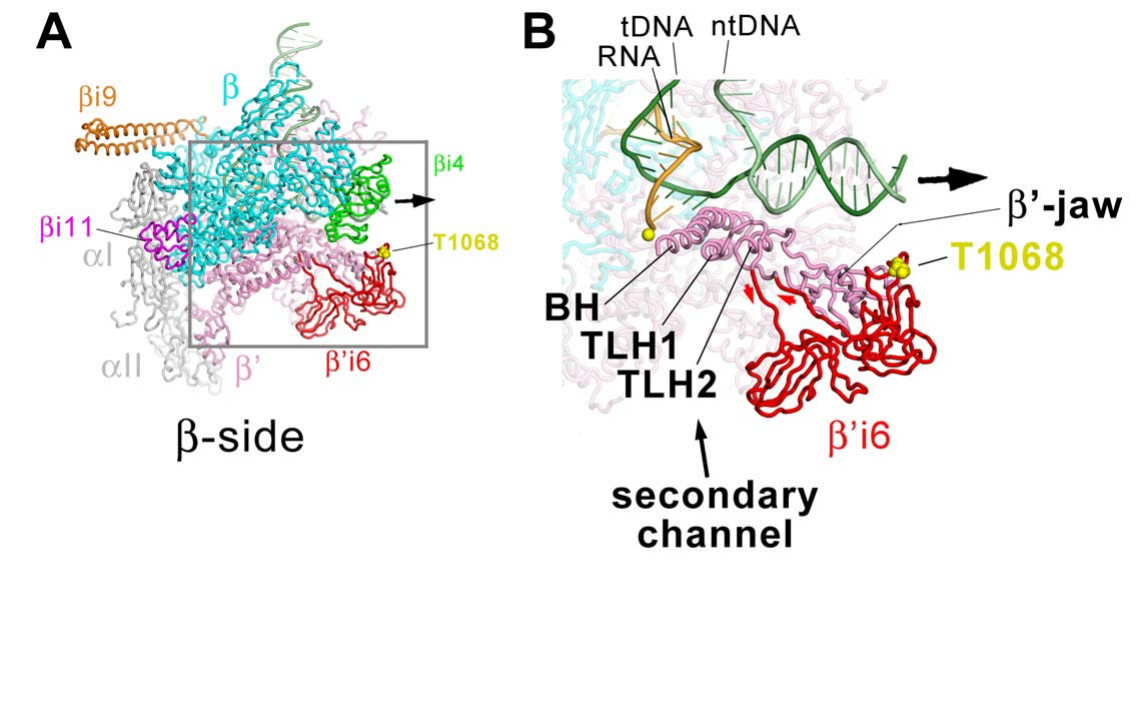
Structural context of *Eco* β'i6. (A) β-side view of the *Eco* RNAP TEC model. The RNAP is shown as a backbone worm (αI, αII, grey; β, cyan, except βi4 is green, βi9 is orange, βi11 is magenta; β', pink, except β'i6 is red). β'T1068 (within β'i6), which is phosphorylated by bacteriophage T7 Gp0.7 [48], is shown as yellow CPK atoms. The nucleic acids are shown as phosphate backbone worms (DNA template strand, dark green; DNA nontemplate strand, light green; RNA transcript, gold). The thick black arrow points in the downstream direction. The boxed region is magnified in (B). (B) Magnified view of boxed region from (A). The obscuring portion of the β subunit has been removed to reveal the inside surface of the RNAP active site channel. Color-coding is the same as (A) but the BH, TLH1, TLH2, the β'-jaw, and β'i6 are highlighted. The active-site Mg^2+^-ion is shown as a yellow sphere.

These profound effects of β'i6 on *Eco* RNAP function are likely due to its insertion in the middle of a critical and highly conserved structural feature of the RNAP, the so-called “trigger-loop” (TL), which connects two highly conserved α-helices (TL-helices 1 and 2, TLH1 and TLH2; Figures 1, 8). The TLHs, in turn, interact with another central structural element, the bridge-helix (BH; Figure 8B). The TL tends to be unstructured (open) in RNAP and in the substrate-free TEC but is found in a structured conformation (closed) where it makes many direct contacts with the incoming NTP substrate in the TEC [38],[39]. The TL has been proposed to cycle between open and closed conformations at each nucleotide addition step to promote rNTP substrate recognition, enzyme fidelity, and possibly catalysis [38]–[42].

Microcin J25 (MccJ25) is a bactericidal 21-residue peptide that inhibits transcription by binding bacterial RNAP within the secondary channel [43]–[46]. Based on saturation mutagenesis of *Eco rpoC* (the gene encoding the RNAP β' subunit), MccJ25 does not contact β'i6; most amino acid substitutions that yield strong resistance against MccJ25 lie in the BH and the TL [43],[44],[46]. Nevertheless, a deletion of β'i6 perturbs the effects of MccJ25 [46], likely through the effects of the β'i6 deletion on the TL conformation.

Our positioning of β'i6 in the spEM density (Figures 4, S5, S6) and its connections with the open TL conformation (Figure 8B) are similar to the results of Hudson et al. [15]. The β'i6 sits outside the RNAP active site channel and makes extensive interactions with the β'-jaw (Figure 8B). The N-terminal SBHM domain of β'i6 (SBHMa) faces the secondary channel, consistent with the results of crosslinks mapped from backtracked TECs (in which the 3′-end of the RNA transcript is extruded out the secondary channel) between analogs incorporated into the RNA 3′-end and the N-terminal region of β'i6 [28]. SBHMb faces the downstream double-stranded DNA-binding channel (Figures 5, 8) but does not contact the DNA; the closest approach between the DNA and β'i6 is 16 Å (between β'D1073 and the nontemplate strand backbone phosphate at +14). Moreover, β'i6 is highly acidic over its entire solvent-exposed surface, including the region facing the downstream double-stranded DNA (Figure 5, front view).

Although β'i6 connects readily to the open conformation of the TL via extended linkers (Figure 8B), modeling suggests it would not be able to connect with the closed TL conformation in the modeled position, a conclusion also reached by Hudson et al. [15]. Since the folding of the TL is required for interactions between highly conserved TL-residues and the incoming nucleotide substrate [19],[38],[39], it is likely that the position of β'i6 must change to accommodate the folded TL conformation at each nucleotide addition step of the transcription cycle.

During bacteriophage T7 infection, the *Eco* RNAP β' subunit is phosphorylated by the phage-encoded kinase Gp0.7 [47], and the site of phosphorylation has been identified as a single amino acid in β'i6, T1068 (Figures 5, 8) [48]. Phosphorylation at this site appears to affect pausing, as well as ρ-dependent termination behavior, of *Eco* RNAP [48]. This site is in the β'i6 loop that makes the closest approach to the downstream DNA, but as discussed above, this region is nevertheless not in close contact with the DNA. The surface is already overall acidic (Figure 5, front view), so it seems unlikely that phosphorylation at this site affects RNAP function by affecting interactions with the downstream DNA.

### Conclusions

An understanding of the basic principles of transcription and its regulation has been garnered largely through detailed study of the transcription system of one organism, *Eco*, which has served as a model for understanding transcription at the molecular and cellular level for more than four decades. The detailed and comprehensive structural description of *Eco* core RNAP and an *Eco* RNAP TEC presented here sheds new light on the interpretation of previous biochemical and genetic data. Moreover, the molecular models provide a structural framework for designing future experiments to investigate the function of the *Eco* RNAP lineage-specific insertions and their role in the *Eco* transcription program, allowing a fuller exploitation of *Eco* as a model transcription system.

## Materials and Methods

### Crystallization and Structure Determination of *Eco* RNAP β2-βi4

*Eco* β2-βi4 was amplified by the polymerase chain reaction from the *Eco rpoB* expression plasmid pRL706 [49] and cloned between the NdeI and BamHI sites of a pET28a-based expression plasmid, creating pSKB2(10-His)*Eco*β2-βi4, encoding *Eco* β2-βi4 with an N-terminal PreScission protease (GE Healthcare) cleavable His_10_-tag. The pSKB2(10-His)*Eco*β2-βi4 was transformed into *Eco* BL21 (DE3) cells. After growing transformed cells in LB medium with kanamycin (50 µg/ml) at 37 °C to an A_600 nm_ = 0.6, isopropyl β-D-1-thiogalactopyranoside was added to a final concentration of 1 mM and cells were grown for an additional 3 h at 37 °C. Cells were harvested by centrifugation, resuspended in lysis buffer (20 mM Tris-HCl, 0.5 M NaCl, 0.5 mM β-mercaptoethanol, 5% v/v glycerol, 0.5 mM phenylmethanesulphonylfluoride), lysed in a continuous-flow French press (Avestin), and clarified by centrifugation. The protein was purified by HiTrap Ni^2+^-chelating affinity chromatography (GE Healthcare) and the His_10_-tag was removed using PreScission protease (GE Healthcare). The sample was further purified by a second, subtractive HiTrap Ni^2+^-chelating affinity chromatography step to remove uncleaved His_10_-tagged protein and the His_10_-tag released from the cleaved product, and gel filtration chromatography (Superdex 75, GE Healthcare). The purified protein was concentrated to 17 mg/ml by centrifugal filtration (VivaScience) and exchanged into storage buffer (10 mM Tris-HCl, pH 8.0, 0.15 M NaCl, 1 mM DTT), and stored at –80 °C. Selenomethionyl-substituted protein was prepared by suppression of methionine biosynthesis [50] and purified by using similar procedures. Reductive methylation of lysine residues was performed as described [20].

Crystals were grown at 22°C in sitting drops using vapor diffusion by mixing equal volumes of protein solution (0.5 µl at 6 mg/ml in storage buffer) and crystallization solution (0.2 M potassium-sodium tartrate, 20% PEG3350). Crystals (irregular plates) appeared after a few days and grew to a maximum size of about 200×100×50 µm in 1 wk. Crystals were prepared for cryo-crystallography by a quick soak in cryo-solution (0.2 M potassium-sodium tartrate, 35% PEG3350), then flash frozen and stored in liquid nitrogen. Diffraction data were collected at beamline X3A at the National Synchrotron Light Source (NSLS, Brookhaven, NY) and processed using HKL2000 [51]. Six of seven possible Se sites were located within the asymmetric unit using the anomalous signal from the Se1 dataset (Table 1) using SHELX [52]. Heavy atom refinement, phasing, and density modification calculations were performed with SHARP [53] using the single-wavelength anomalous dispersion data to 1.9 Å-resolution from the Se1 dataset, as well as the 1.6 Å-resolution Se2 dataset (Table 1), yielding an excellent map that allowed automatic building of almost the entire structure using ARP/wARP [54]. Iterative cycles of refinement and model building were carried out using Coot [55] and RefMac5 [56]. The final model was refined to an *R*/*R*_free_ of 0.209/229 at 1.6 Å-resolution (*R*_free_ was calculated using 5% random data omitted from the refinement). 97.5% of residues fall in the most favored regions of the Ramachandran plot, while no residues are in disallowed regions.

### Crystallization and Structure Determination of *Eco* RNAP βflap-βi9

The *Eco* βflap-βi9 (*Eco* β residues 831–1057) was co-expressed with bacteriophage T4 gp33 [57] as a single operon from a modified pET29a vector [58] and the complex was purified using standard procedures (K.-A.F.T., P. Deighan, S. Nechaev, A. Hochschild, E.P. Geiduschek, S.A.D., in preparation). Selenomethionyl-substituted complex was produced by suppression of methionine biosynthesis [50].

Crystals of the complex were grown at 22°C in sitting drops using vapor diffusion by mixing equal volumes of protein solution (1 µl at 7.5–12 mg/ml in 10 mM Tris-HCl, pH 8.0, 150 mM NaCl, 1% v/v glycerol, 1 mM β-mercaptoethanol, 1 mM DTT) and crystallization solution (0.2 M tri-potassium citrate, 20% w/v PEG3350). Crystals were prepared for cryo-crystallography by slow exchange into cryo-solution (0.2 M tri-potassium citrate, 20% w/v PEG3350, 20% v/v ethylene glycol), then flash frozen and stored in liquid nitrogen. Diffraction data were collected at beamline X3A at the NSLS (Brookhaven, NY) and processed using HKL2000 (Table S1) [51]. A molecular replacement solution was obtained using the Native amplitudes (Table S1) with a search model consisting of a homology model of the *Eco* βflap based on the *Taq* βflap generated using MODELLER (the search model excluded the flexible flap-tip) [59]. The molecular replacement phases were used to locate four Se sites from the anomalous signal of the Se dataset (Table S1). Heavy atom refinement, phasing, and density modification calculations were performed with SHARP [53] using the single-wavelength anomalous dispersion data from the Se dataset (Table S1) yielding an interpretable map (Figure S3). Iterative cycles of refinement and model building were carried out using Coot [55] and RefMac5 [56]. The final model was refined to an *R*/*R*_free_ of 0.265/0.291 at 3.0 Å-resolution (*R*_free_ was calculated using 5% random data omitted from the refinement). 95.25% of residues fall in the most favored regions of the Ramachandran plot, while no residues are in disallowed regions.

### Cryo-EM Reconstruction of *Eco* RNAP by Single-Particle Averaging

Purification of *Eco* core RNAP from an overexpression system was performed as described [60]. This results in highly pure *Eco* RNAP core enzyme, which is deficient in the ω subunit. *Eco* RNAP holoenzyme was prepared by incubating core RNAP (3 mg/ml in 10 mM Tris-HCl, pH 8, 0.2 M NaCl, 0.1 mM EDTA, 5 mM DTT) with a 5-fold molar excess of σ^70^ for 30 min at room temperature. For cryo-EM, a 5 µl sample (0.1 mg/ml in the same buffer) was applied to a Quantifoil grid coated with holey carbon film previously made hydrophilic by glow-discharge. The grid was blotted with filter paper and then immediately plunged into liquid ethane slush. The sample was imaged at 50,000× magnification with a Tecnai F20 transmission electron microscope operating at 200 kV. Micrographs displaying minimal astigmatism were digitized at a 14 µm interval (corresponding to 2.8 Å on the image) using a Zeiss SCAI flat-bed densitometer (ZI/Carl Zeiss). Individual particles were selected by eye and windowed in 90×90 pixel images. Defocus values were estimated from digitized micrographs using ctfit (EMAN) [61].

We generated a spEM reconstruction of *Eco* RNAP by analyzing ∼42,000 cryo-images of *Eco* RNAP particles (Figures 4A, S4–S6). Particle image orientation parameters were approximately determined using reference projections of a volume generated by low-pass filtration of the *Taq* core RNAP X-ray structure [5] to 35 Å-resolution. We used a previously devised protocol in which image orientation parameters are iteratively refined by cycling through sets comprising relatively small numbers of reference projections [62]. After a large number of iterations (130) using the SPIDER software package [63], we obtained a structure in which well-defined densities not present in the original model volume were apparent. Further refinement of image orientation parameters by projection matching [64] using the SPARX software package [25] yielded a structure of *Eco* core RNAP with a 0.5 Fourier-shell cutoff resolution of about 11.2 Å (Figure S4). For further analysis, the map was Fourier filtered using an ahyperbolic tangent low-pass filter [24] as implemented in the SPARX software package [25] with a stop-band frequency of 0.28 and a fall-off of 0.45.

### Sequence Alignments

Alignments for the *Eco* lineage-specific insertions (see Datasets S1–S3) were created using the bacterial lineage-specific insertions alignments from Lane et al. [3] as a starting point. The final alignments were created by iterative cycles in which sequences that did not match the *Eco* domains were removed, followed by re-alignment with MUSCLE [65] or PCMA [66].

### Accession Numbers

Electron Microscopy Data Bank: The single-particle cryoEM reconstruction volume has been deposited under ID code EMD-5169. Protein Data Bank: Atomic coordinates and structure factors for *Eco* RNAP β2-βi4 have been deposited under accession code 3LTI. The EM-fitted coordinate model of *Eco* core RNAP has been deposited under accession code 3LU0. The coordinates of the *Eco* RNAP TEC model are available in the Supporting Information (Dataset S4).

## Supporting Information

Dataset S1 **beta-i9_blast_to_fas_to_aln_man4_cull.msf – Sequence alignment (msf format) containing 307 non-redundant βi9 sequences.**
(0.07 MB TDS)

Dataset S2 **beta-i4_blast_to_fas_to_aln_man5_cull.msf – Sequence alignment (msf format) containing 316 non-redundant βi4 sequences (only** ***Eco*** **-like βi4 sequences comprising two BBM2 domains).**
(0.12 MB TDS)

Dataset S3 **beta-i11_blast_to_fas_to_aln_man4_cull.msf – Sequence alignment (msf format) containing 310 non-redundant βi11 sequences.**
(0.07 MB TDS)

Dataset S4 **Eco_TEC_model.pdb – Coordinates (PDB format) of the** ***Eco*** **TEC model.**
(2.22 MB TXT)

Figure S1 ***Eco*** **β2-βi4 electron density map.** Stereo view of the 1.6 Å-resolution 2|*F*_o_|–|*F*_c_| map, contoured at 1.5 σ. The model is shown as sticks, with nitrogen atoms colored blue, oxygen atoms red, and carbon atoms colored according to Figure 2B. Water molecules are represented as red spheres. Shown is the region surrounding dimethylated [20] K324.
(2.07 MB TIF)

Figure S2 **Comparison of** ***Taq*** **β2 and** ***Eco*** **β2-βi4.** The two structures were superimposed over 100 α-carbon positions (excluding flexible loops connecting secondary structural elements), yielding a root-mean-square-deviation of 1.68 Å. Other than the insertion of βi4 in *Eco*, significant differences in the β2 structures include: (i) the loop connecting the first two β-strands of the β2 domain, where *Eco* has a 5-residue insertion (*Eco* β residues 164–168, disordered in the structure), and (ii) the loop connecting the last two α-helices of the β2 domain, which includes a 7-residue insertion present in *Taq* β (*Taq* β residues 293–299; Figure 2A).
(5.47 MB TIF)

Figure S3 ***Eco*** **βflap-βi9 electron density map.** Stereo view of the 3.0 Å-resolution 2|*F*_o_|–|*F*_c_| map, contoured at 1.0 σ. The model is shown as sticks, with nitrogen atoms colored blue, oxygen atoms red, and carbon atoms colored according to Figure 3B. Shown is a region of the βi9 ladder helices.
(2.90 MB TIF)

Figure S4 **Image analysis.** (A) Unprocessed electron micrograph of a field of *Eco* RNAP molecules preserved in vitreous ice. Selected particles are circled. (B) Distribution of image orientations, plotted as a polar-angle diagram, viewed along the θ = 0° axis. (C) Fourier shell correlation [67],[68] as a function of spatial frequency.
(1.54 MB TIF)

Figure S5 **Back, bottom, channel, and front views of spEM density and fit of** ***Eco*** **RNAP model.** For each view, the left image shows the spEM density map (grey surface, contoured at 2.5 σ), and the right image shows the spEM density map (grey transparent surface) with the fitted *Eco* RNAP homology model superimposed (excluding ω, the C-terminal 41 residues of β', and βi9). The *Eco* RNAP homology model is shown as a backbone worm, color-coded as in Figure 4.
(7.72 MB TIF)

Figure S6 **β**'**-side, bottom, β-side, and top views of spEM density and fit of** ***Eco*** **RNAP model.** For each view, the left image shows the spEM density map (grey surface, contoured at 2.5 σ), and the right image shows the spEM density map (grey transparent surface) with the fitted *Eco* RNAP homology model superimposed (excluding ω, the C-terminal 41 residues of β', and βi9). The *Eco* RNAP homology model is shown as a backbone worm, color-coded as in Figure 4.
(8.62 MB TIF)

Figure S7 **Structural features of** ***Eco*** **βi9.** Two views of *Eco* βi9 are shown: The left column shows the “front” view (the side facing the “hook”), and the right column shows the “back” view (the side away from the “hook”). The top row shows the backbone ribbon. The middle row shows the structure (with transparent molecular surface) colored in a gradient according to the Blosum 62 information score (as determined by the program PFAAT [70]) calculated from an alignment of 307 non-redundant βi9 sequences (see Supporting Information). The color gradient covers scores from 0 to 1 (0, white; 0.5, yellow; 1.0, red). Individual residues with score ≥0.75 are labeled. Underlined residues denote residues with significant solvent accessibility. The bottom row shows the molecular surface colored according to the electrostatic surface distribution of the solvent-accessible surface in units of kT (−5, red; 0, white; +5, blue), as calculated by APBS [69].
(6.13 MB TIF)

Figure S8 **Details of** ***ab initio*** **-predicted** ***Eco*** **βi11 structure.** (A) Sequence context of *Eco* RNAP βi11. The secondary structure for the predicted *Eco* βi11 structure (determined using the Robetta server (http://robetta.bakerlab.org/)) is indicated directly below the sequence (filled rectangles denote α-helices). Above the number scale, black lines denote the sequence regions common to all bacterial RNAPs [3]. Gaps in the βi11 sequence with numbers above denote the location and residue length of insertions in an alignment of 310 non-redundant βi11 sequences (see Supporting Information). The insertions all occur in loops connecting the helices. The extent of *Eco* βi11 is denoted by the thick magenta line (above). (B) Backbone ribbon of the predicted *Eco* βi11 structure. The grey spheres mark α-carbon positions surrounding the insertions from the sequence alignment. The numbers pointing to each insertion point denote the insertion length. (C) The predicted *Eco* βi11 structure is colored in a gradient according to the Blosum 62 information score (as determined by the program PFAAT [70]) calculated from the alignment of 310 non-redundant βi11 sequences (see Supporting Information). The color gradient covers scores from 0 to 1 (0, white; 0.5, yellow; 1.0, red). Individual residues with score ≥0.75 are labeled. Nearly all of the conserved hydrophobic residues are buried in the hydrophobic core of the structure. Two solvent-accessible polar residues (R1142 and D1166) form an apparently conserved salt-bridge that may stabilize the structure.
(3.18 MB TIF)

Table S1 **Crystallographic statistics for** ***Eco*** **RNAP βflap-βi9 crystals.**
(0.04 MB DOC)

## Abbreviations

**Abbreviations** BBM1: β-β' module 1
BBM2: β-β' module 2
BH: bridge-helix
Cryo-EM: cryo-electron microscopy
*Eco*: *Escherichia coli*
hEM: helical cryo-electron microscopy
MccJ25: microcin J25
NSLS: National Synchrotron Light Source
NYSBC: New York Structural Biology Center
*paf*: prevent Alc function
RNAP: RNA polymerase
RU-SBRC: The Rockefeller University Structural Biology Resource Center
SBHM: sandwich-barrel hybrid motif
spEM: single-particle cryo-electron microscopy
TEC: ternary elongation complex
*Taq*: *Thermus aquaticus*
TL: trigger-loop
TLH1: trigger-loop helix 1
TLH2: trigger-loop helix 2
*Tth*: *Thermus thermophilus*
us-DNA: upstream DNA
