# Supplementary figures and images for: Complete Structural Model of Escherichia coli RNA Polymerase from a Hybrid Approach
Source: PLoS Biol. 2010 Sep 14;8(9):e1000483. doi: 10.1371/journal.pbio.1000483 (PMC2939025; doi:10.1371/journal.pbio.1000483)

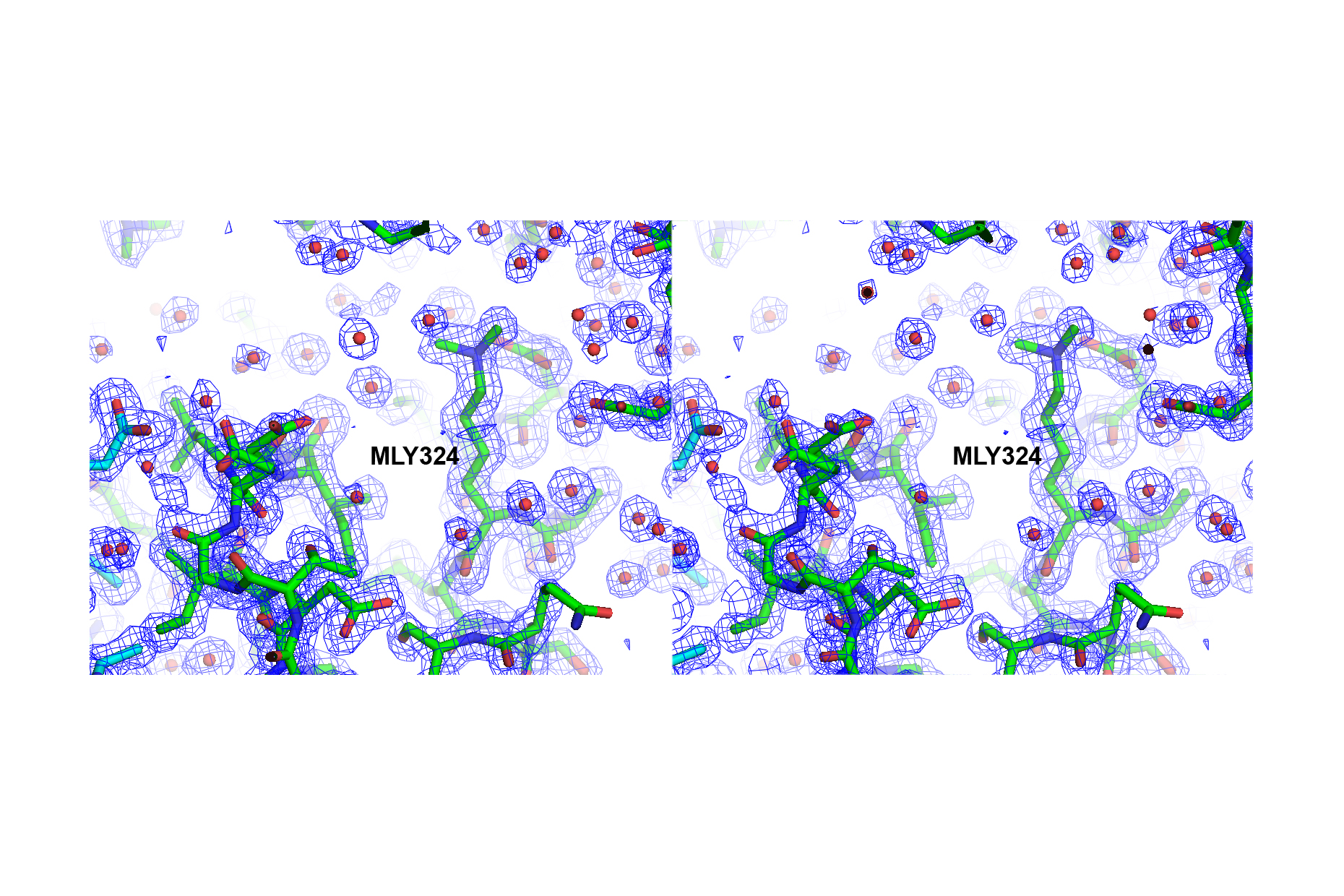

Supplement: Figure S1 — Eco β2-βi4 electron density map. Stereo view of the 1.6 Å-resolution 2|F o|–|F c| map, contoured at 1.5 σ. The model is shown as sticks, with nitrogen atoms colored blue, oxygen atoms red, and carbon atoms colored according to Figure 2B. Water molecules are represented as red spheres. Shown is the region surrounding dimethylated [20] K324. (2.07 MB TIF) [file pbio.1000483.s005.tif]

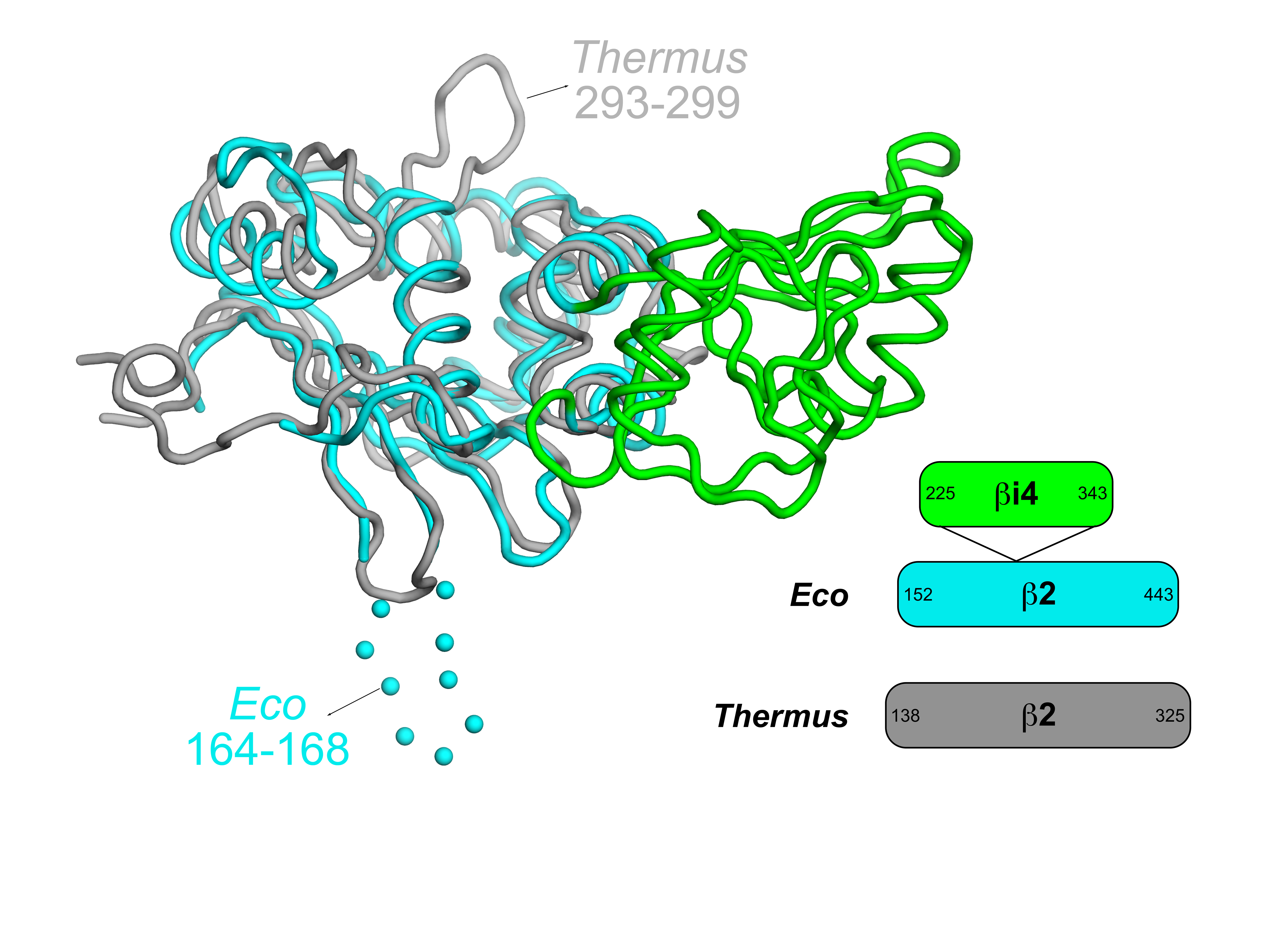

Supplement: Figure S2 — Comparison of Taq β2 and Eco β2-βi4. The two structures were superimposed over 100 α-carbon positions (excluding flexible loops connecting secondary structural elements), yielding a root-mean-square-deviation of 1.68 Å. Other than the insertion of βi4 in Eco, significant differences in the β2 structures include: (i) the loop connecting the first two β-strands of the β2 domain, where Eco has a 5-residue insertion (Eco β residues 164–168, disordered in the structure), and (ii) the loop connecting the last two α-helices of the β2 domain, which includes a 7-residue insertion present in Taq β (Taq β residues 293–299; Figure 2A). (5.47 MB TIF) [file pbio.1000483.s006.tif]

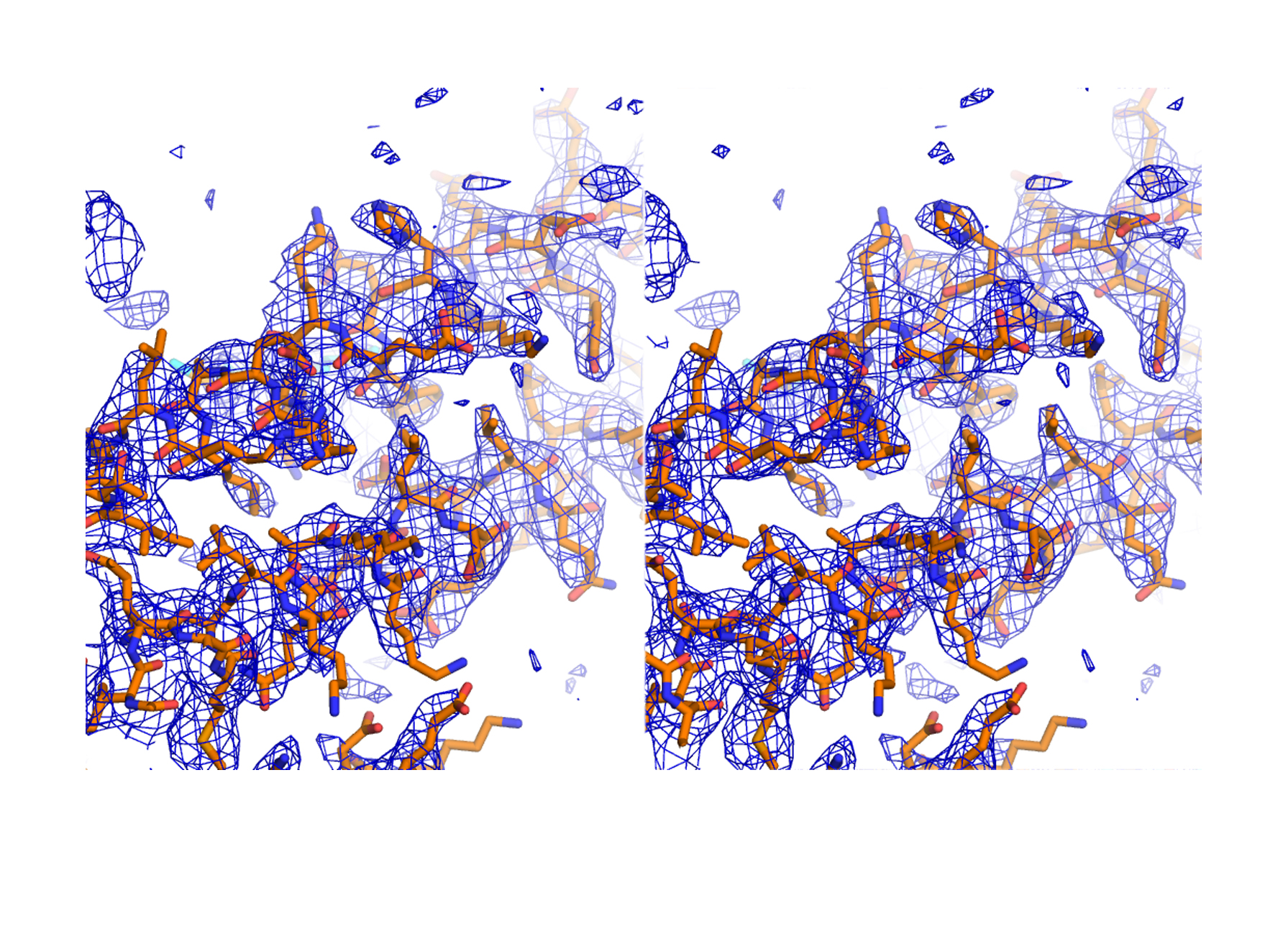

Supplement: Figure S3 — Eco βflap-βi9 electron density map. Stereo view of the 3.0 Å-resolution 2|F o|–|F c| map, contoured at 1.0 σ. The model is shown as sticks, with nitrogen atoms colored blue, oxygen atoms red, and carbon atoms colored according to Figure 3B. Shown is a region of the βi9 ladder helices. (2.90 MB TIF) [file pbio.1000483.s007.tif]

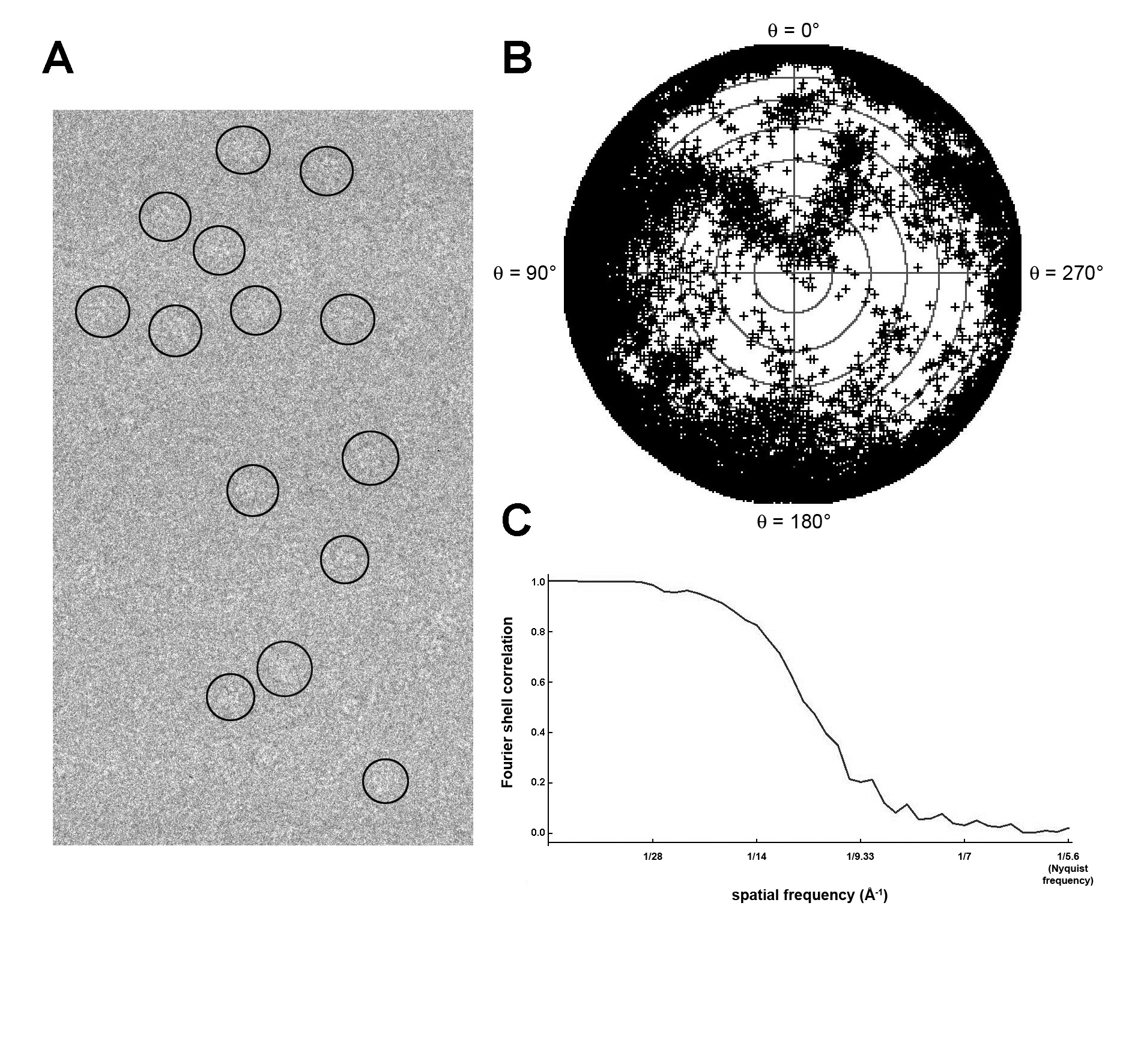

Supplement: Figure S4 — Image analysis. (A) Unprocessed electron micrograph of a field of Eco RNAP molecules preserved in vitreous ice. Selected particles are circled. (B) Distribution of image orientations, plotted as a polar-angle diagram, viewed along the θ = 0° axis. (C) Fourier shell correlation [67],[68] as a function of spatial frequency. (1.54 MB TIF) [file pbio.1000483.s008.tif]

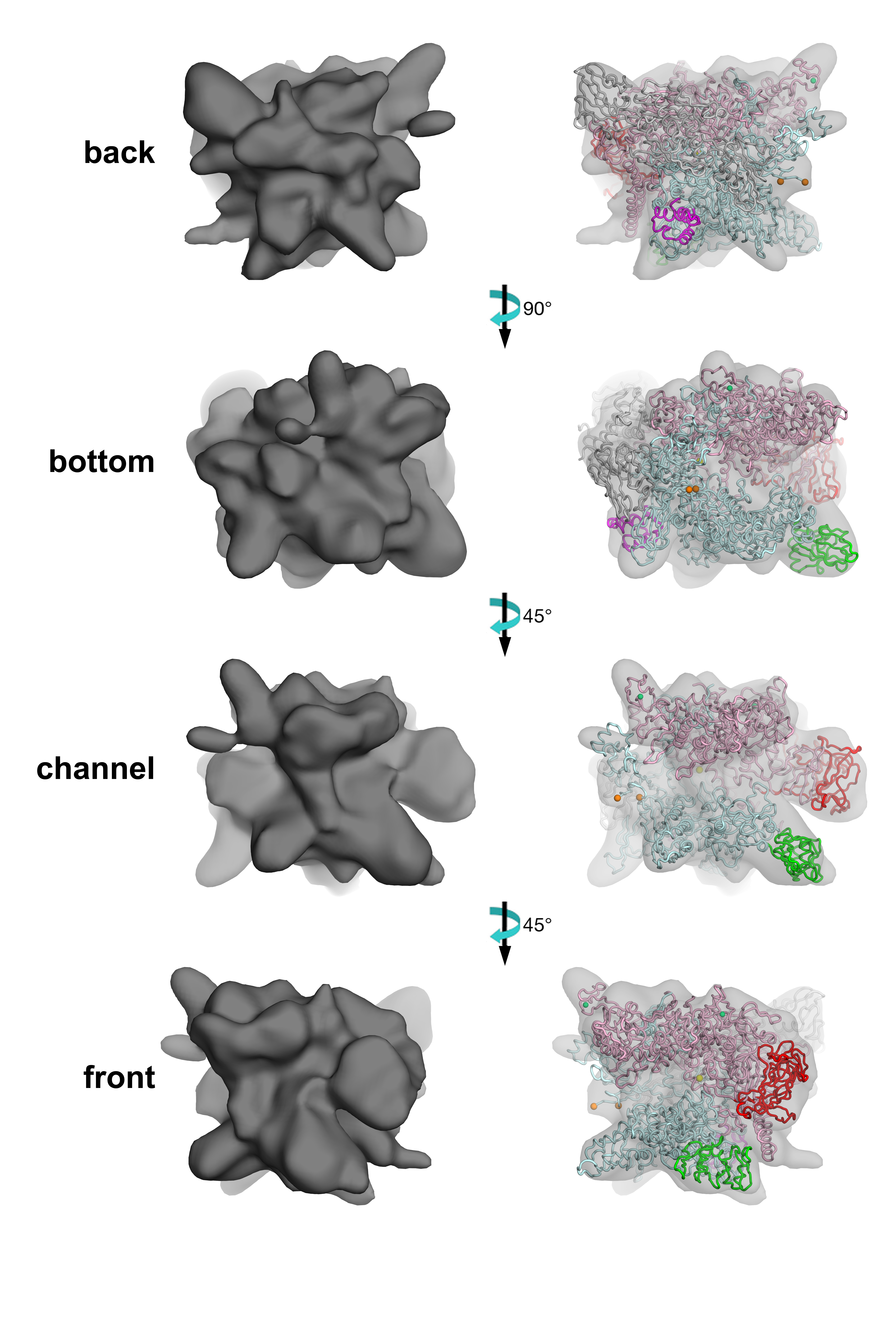

Supplement: Figure S5 — Back, bottom, channel, and front views of spEM density and fit of Eco RNAP model. For each view, the left image shows the spEM density map (grey surface, contoured at 2.5 σ), and the right image shows the spEM density map (grey transparent surface) with the fitted Eco RNAP homology model superimposed (excluding ω, the C-terminal 41 residues of β', and βi9). The Eco RNAP homology model is shown as a backbone worm, color-coded as in Figure 4. (7.72 MB TIF) [file pbio.1000483.s009.tif]

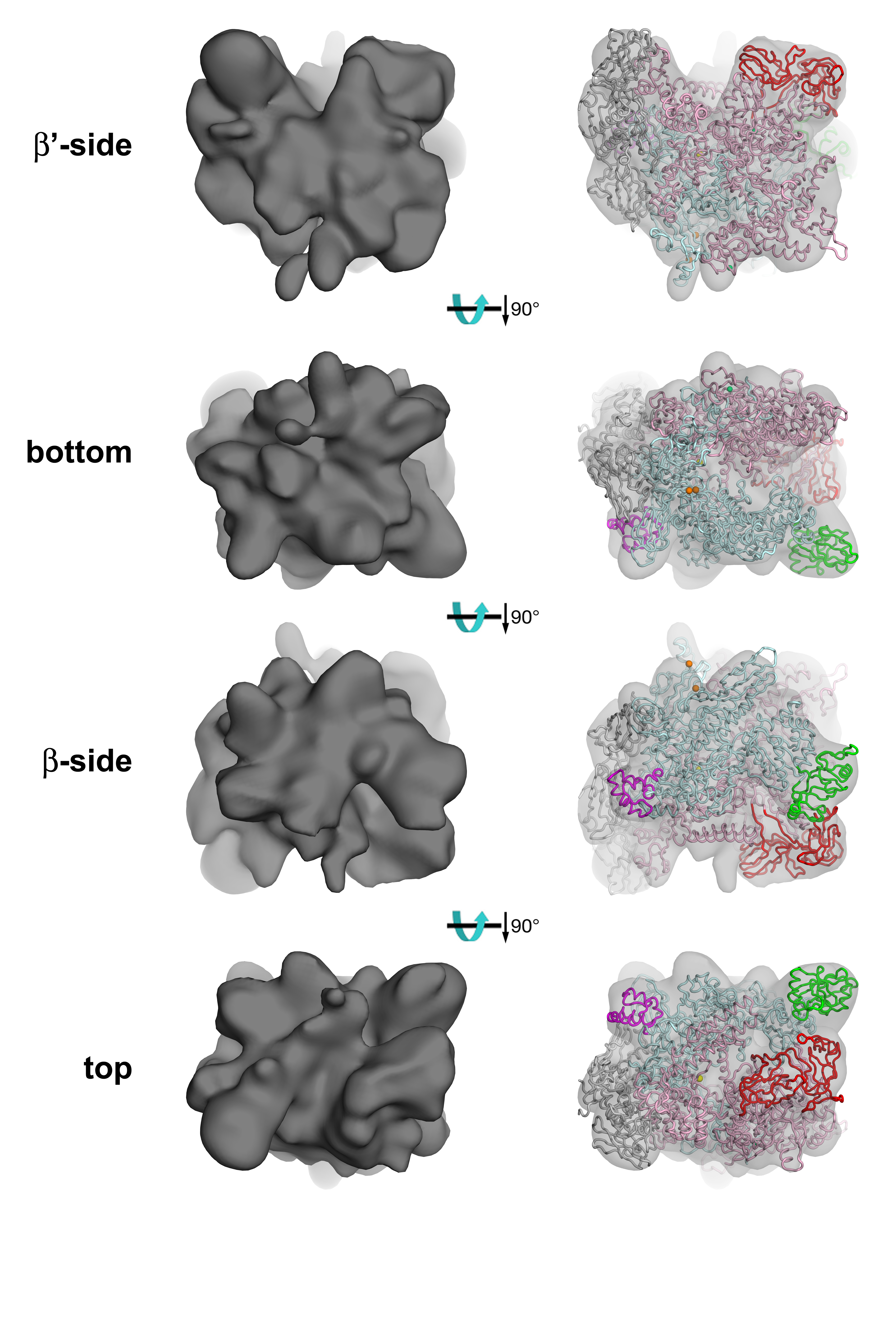

Supplement: Figure S6 — β'-side, bottom, β-side, and top views of spEM density and fit of Eco RNAP model. For each view, the left image shows the spEM density map (grey surface, contoured at 2.5 σ), and the right image shows the spEM density map (grey transparent surface) with the fitted Eco RNAP homology model superimposed (excluding ω, the C-terminal 41 residues of β', and βi9). The Eco RNAP homology model is shown as a backbone worm, color-coded as in Figure 4. (8.62 MB TIF) [file pbio.1000483.s010.tif]

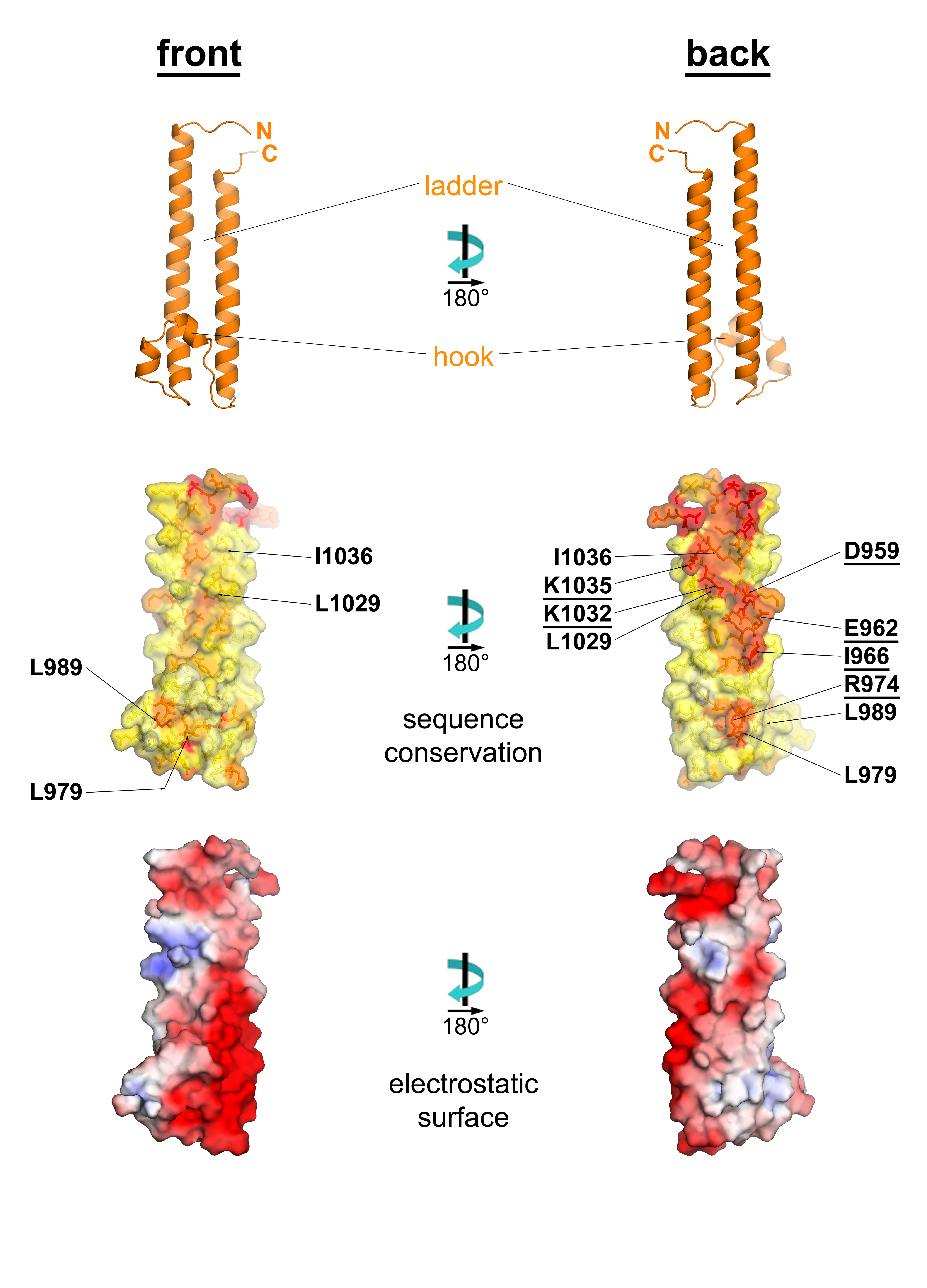

Supplement: Figure S7 — Structural features of Eco βi9. Two views of Eco βi9 are shown: The left column shows the “front” view (the side facing the “hook”), and the right column shows the “back” view (the side away from the “hook”). The top row shows the backbone ribbon. The middle row shows the structure (with transparent molecular surface) colored in a gradient according to the Blosum 62 information score (as determined by the program PFAAT [70]) calculated from an alignment of 307 non-redundant βi9 sequences (see Supporting Information). The color gradient covers scores from 0 to 1 (0, white; 0.5, yellow; 1.0, red). Individual residues with score ≥0.75 are labeled. Underlined residues denote residues with significant solvent accessibility. The bottom row shows the molecular surface colored according to the electrostatic surface distribution of the solvent-accessible surface in units of kT (−5, red; 0, white; +5, blue), as calculated by APBS [69]. (6.13 MB TIF) [file pbio.1000483.s011.tif]

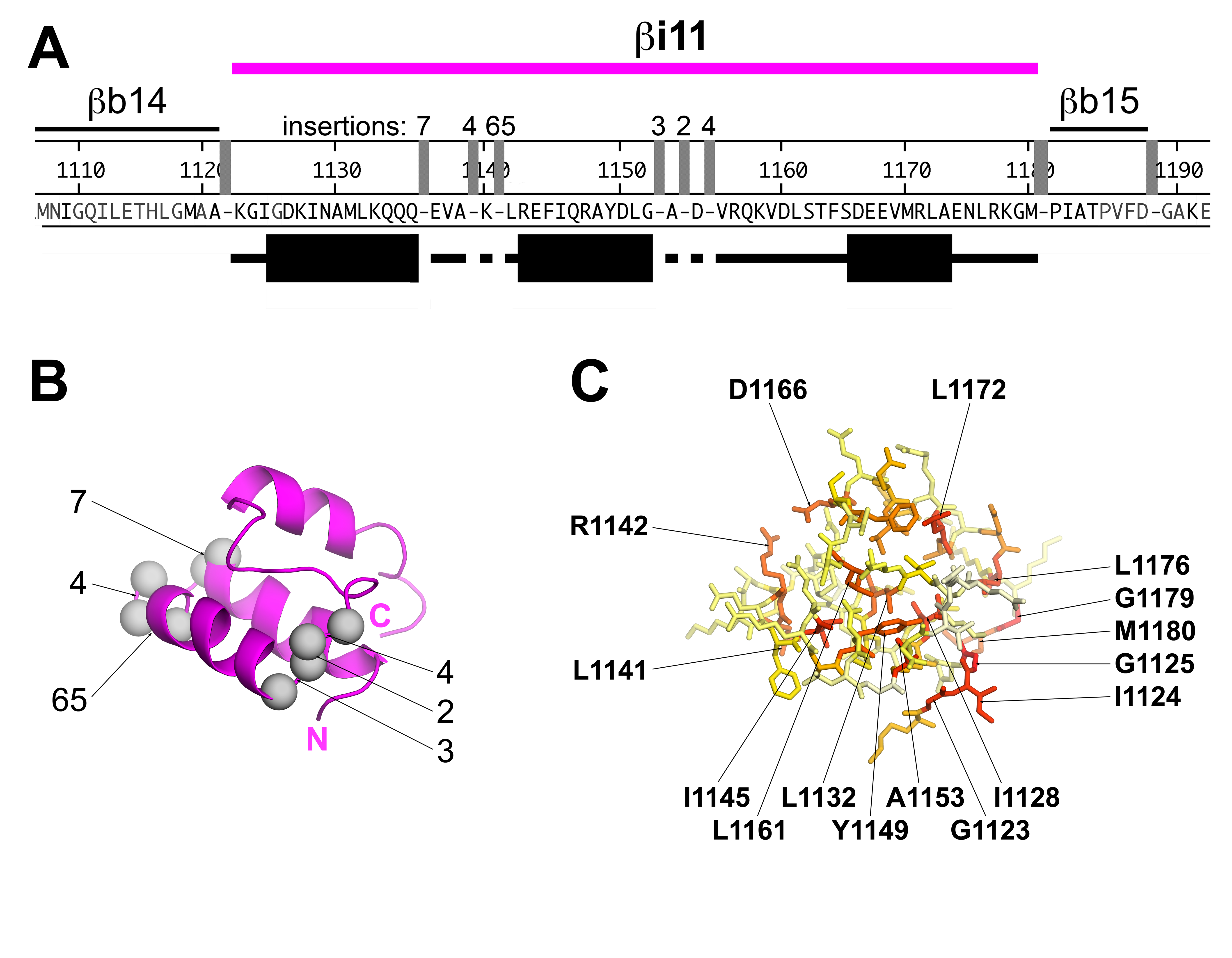

Supplement: Figure S8 — Details of ab initio -predicted Eco βi11 structure. (A) Sequence context of Eco RNAP βi11. The secondary structure for the predicted Eco βi11 structure (determined using the Robetta server (http://robetta.bakerlab.org/)) is indicated directly below the sequence (filled rectangles denote α-helices). Above the number scale, black lines denote the sequence regions common to all bacterial RNAPs [3]. Gaps in the βi11 sequence with numbers above denote the location and residue length of insertions in an alignment of 310 non-redundant βi11 sequences (see Supporting Information). The insertions all occur in loops connecting the helices. The extent of Eco βi11 is denoted by the thick magenta line (above). (B) Backbone ribbon of the predicted Eco βi11 structure. The grey spheres mark α-carbon positions surrounding the insertions from the sequence alignment. The numbers pointing to each insertion point denote the insertion length. (C) The predicted Eco βi11 structure is colored in a gradient according to the Blosum 62 information score (as determined by the program PFAAT [70]) calculated from the alignment of 310 non-redundant βi11 sequences (see Supporting Information). The color gradient covers scores from 0 to 1 (0, white; 0.5, yellow; 1.0, red). Individual residues with score ≥0.75 are labeled. Nearly all of the conserved hydrophobic residues are buried in the hydrophobic core of the structure. Two solvent-accessible polar residues (R1142 and D1166) form an apparently conserved salt-bridge that may stabilize the structure. (3.18 MB TIF) [file pbio.1000483.s012.tif]
