# Supplementary material for: Complete Structural Model of Escherichia coli RNA Polymerase from a Hybrid Approach
Source: PLoS Biol. 2010 Sep 14;8(9):e1000483. doi: 10.1371/journal.pbio.1000483 (PMC2939025; doi:10.1371/journal.pbio.1000483)
Supplement: Table S1 — Crystallographic statistics for Eco RNAP βflap-βi9 crystals. (0.04 MB DOC) [file pbio.1000483.s013.doc]

**Table S1** Crystallographic statistics for *Eco* RNAP βflap-βi9 crystals.

|  | Nat | Se^a^ |
| --- | --- | --- |
| **Data collection** |  |  |
| Space group | I222 | I222 |
| Cell dimensions   |  |  |
| *a*, *b*, *c* (Å) | 54.70, 110.19, 164.72 | 55.22, 112.25, 164.85 |
|     α, β, γ  (°) | 90, 90, 90 | 90, 90, 90 |
|  |  | *Peak* |
| Wavelength | 0.9785 | 0.9785 |
| Resolution (Å) | 30.0-3.00 (3.11-3.00) | 30.0-3.00 (3.11-3.00) |
| *R*_sym_ | 0.058 (0.57) | 0.053 (0.412) |
| *I* / σ*I* | 25.0 (1.7) | 21.9 (2.6) |
| Completeness (%) | 89.8 (79.8) | 98.1 (93.6) |
| Redundancy | 4.2 (3.0) | 3.0 (2.7) |
|  |  |  |
| **Refinement** |  |  |
| Resolution (Å) |  | 30.0-3.00 |
| No. reflections |  | 9,980 |
| *R*_work_ / *R*_free_ |  | 0.264/0.291 |
| No. atoms |  |  |
| Protein |  | 2,325 |
| Water |  | 0 |
| *B*-factors |  |  |
| Protein |  | 29.34 |
| R.m.s deviations |  |  |
| Bond lengths (Å) |  | 0.008 |
| Bond angles (°) |  | 1.108 |

^a^Scaling statistics for Se1 dataset calculated without combining anomalous pairs
